# Use and Utility of Hemostatic Screening in Adults Undergoing Elective, Non-Cardiac Surgery

**DOI:** 10.1371/journal.pone.0139139

**Published:** 2015-12-01

**Authors:** Isabel A. Weil, Sinziana Seicean, Duncan Neuhauser, Nicholas K. Schiltz, Andreea Seicean

**Affiliations:** 1 Department of Epidemiology and Biostatistics, Case Western Reserve University, Cleveland, Ohio, United States of America; 2 Heart and Vascular Institute, Cleveland Clinic, Cleveland, Ohio, United States of America; 3 Departments of Pulmonary, Critical Care and Sleep Medicine, University Hospitals, Cleveland, Ohio, United States of America; University of Modena & Reggio Emilia, ITALY

## Abstract

**Introduction:**

One view of value in medicine is outcome relative to cost of care provided. With respect to operative care, increased attention has been placed on evaluation and optimization of patients prior to undergoing an elective surgery. We examined more than 2 million patients having elective, non-cardiac surgery to assess the incidence and utility of pre-operative hemostatic screening, compared with a composite of history variables that may indicate a propensity for bleeding, to assess several important outcomes of surgery.

**Materials & Methods:**

We queried the NSQIP database to identify 2,020,533 patients and compared hemostatic tests (PT, aPTT, platelet count) and history covariables indicative of potential for abnormal hemostasis. We compared outcomes across predictor values; used Person’s chi-square tests to compare differences, and logistic regression to model outcomes.

**Results:**

Approximately 36% of patients had all three tests pre-operatively while 16% had none of them; 11.2% had a history predictive of potential abnormal bleeding. Outcomes of interest across the cohort included death in 0.7%, unplanned return to the operating room or re-admission within 30 days in 3.8% and 6.2% of patients; 5.3% received a transfusion during or after surgery. Sub-analyses in each of the nine surgical specialties’ most common procedures yielded similar results.

**Conclusion:**

The limited predictive value of each hemostatic screening test, as well as excess costs associated with them, across a broad spectrum of elective surgeries, suggests that limiting pre-operative testing to a more select group of patients may be reasonable, equally efficacious, efficient, and cost-effective.

## Introduction

Nearly 30% of the greater than 38 million hospitalizations in the United States yearly involve a surgical procedure^1–6^. Hospital stays that include an operation result in length of stays nearly twice as long as those without a surgical procedure and stays with an operation accounted for nearly half of the $377 billion in hospital costs accumulated in 2012 [1–6]. Given that the majority of these surgical hospitalizations involve an elective procedure routine, outpatient, pre-operative evaluation is increasingly important to the medical optimization of the surgical patient [2,6].

In light of the evolving nature of elective surgery, increased attention has been placed on pre-operative evaluation. One example is a renewed focus on the utility of routine laboratory assessment to guide or predict peri-operative care and outcomes. Several assessments from the United Kingdom and the United States, for example, suggest that universal pre-operative screening of the aPTT, PT/INR, or the platelet count, may be excessive or unnecessary [7–11]. As part of the *Choosing Wisely* campaign, the American Society of Anesthesiologists (ASA) and the American Society of Clinical Pathology have both advised against routine pre-operative testing for low risk surgery [12–13]. While the former group suggested low risk meant ASA class 1 and I2 patients, the latter^13^ was less specific, yet noted: “in almost all cases, no adverse outcomes are observed when clinically stable patients undergo elective surgery, irrespective of the whether an abnormal test is identified.”

We examined a large population of patients undergoing elective, non-cardiac surgery, across a spectrum of surgical disciplines and levels of operative risk, to assess several questions. First, we sought to determine the incidence of hemostatic screening—and the frequency of abnormal results—immediately prior to an elective surgery in a large, multi-center sample of US adults over a recent, seven-year period (2006–2012). Second, we wished to compare routine tests of hemostasis with a composite of patient history variables and the ability of one, the other, or both to predict peri-operative bleeding and a few, important outcomes of surgery. Finally, we wished to assess whether laboratory testing or patient variables may indicate dysfunctional hemostasis as an harbinger of peri-operative complications and outcome after elective surgery and whether this differs between surgical conditions.

## Methods

### Data Source

This study evaluated the medical records of all patients who underwent elective, non-cardiac surgery included in the American College of Surgeon (ACS) National Surgical Quality Improvement Program (NSQIP) database between 2006 and 2012. Detailed description of the ACS-NSQIP database, including design, sampling strategy, and variable definitions can be found elsewhere [14–15]. All data was collected prospectively using a standardized protocol, which consists of strictly defined variables. Each participating site had a trained, surgical nurse reviewer responsible for accurate and reproducible data collection from computerized and paper patient medical records, physician office records, and telephone interviews with the patient. The ACS-NSQIP database has been validated for accuracy and reproducibility [14–16]. This study was approved by the Cleveland Clinic Institutional Review Board (IRB#8482). The NSQIP database data is anonymous and de-identified prior being released to researchers by the ACS.

### Subjects

**Fig 1:**
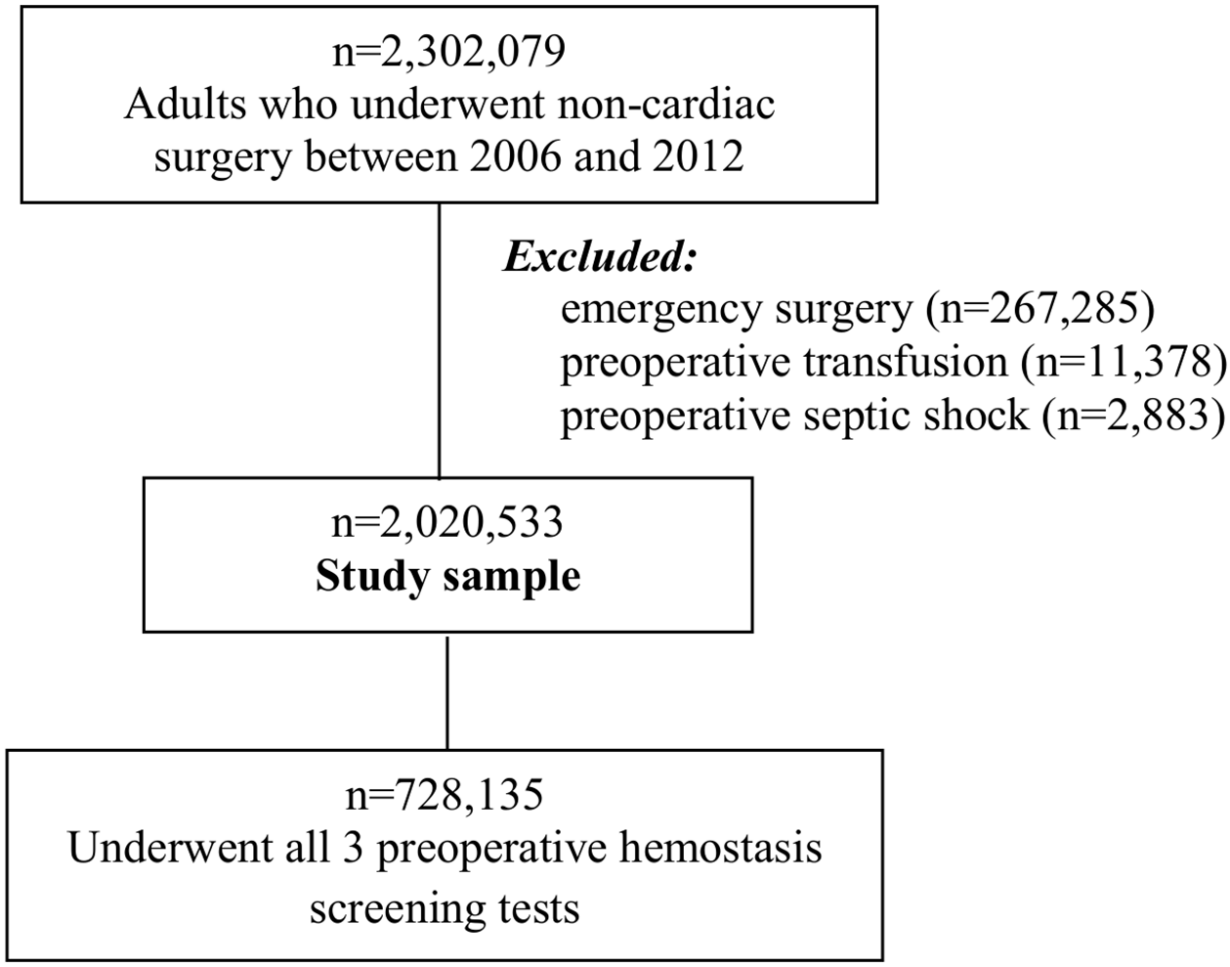
Patient selection criteria. A schematic of the patients included and excluded in this study from the entire population in NSQIP (n = 2,301,079).

We identified 2,302,079 adult patients who underwent an elective, non-cardiac surgery between 2006 and 2012 (Fig 1). We excluded patients undergoing an emergency operation (n = 267,285), patients with sepsis (n = 2,883), and those who received preoperative transfusion (n = 11,378) [17]. Our final study sample contained 2,020,533 patients (Fig 1). We excluded patients undergoing cardiac surgery for several reasons: because the nature of open heart surgery, including cardiac bypass, increases coagulation disturbances and enhances platelet activation, among other features, and which therefore alters the utility function of pre-operative hemostatic screening; and, since up to 50% of patients receive an allogeneic blood transfusion [18–19].

Sub-analyses by surgical specialty were conducted as well and are reported separately (please see S1 Text). To ensure that our findings in the sub-analyses would be most relevant to each subspecialty, we chose to limit our samples, in each surgical specialty, to patients who had the 10 most common diagnoses, which we determined using International Classification of Disease (ICD-9) codes and who underwent the 10 most common surgeries, according to primary Current Procedure Terminology codes (see S1 Text).

### Hemostatic screening lab values—(predictor variables)

Preoperative hemostatic screening lab values were recorded in the NSQIP database if drawn within 90 days prior to the surgical procedure; NSQIP records the laboratory value drawn closest temporally to the index surgery. An abnormal PT was defined as an international normalized ratio (INR) of >1.2, and severely abnormal PT as INR >3.0, as described elsewhere [20–26]. The activated partial thromboplastin time (aPTT) was defined as abnormal if >35 seconds, and as severely abnormal if >60 seconds [20–23]. A low platelet count was defined as <150, 000 cells per microliter (μl) and elevated beyond normal if the platelet count was >450,000 cells per μl [20–23]. Sensitivity analyses were conducted by examining test values as continuous variables and using different cutoff values for abnormal results for all three tests.

### Patient History Covariates—(predictor variables)

We incorporated all history-based risk factors for abnormal hemostasis, as previously identified [24–31]. Self-reported patient history of abnormal bleeding, self-reported family history of bleeding disorders, vitamin K deficiency, and a comprehensive list of medications or medication classes that pose a risk for disordered hemostasis were captured through the NSQIP variable “bleeding disorders” [29]. “Patients requiring regular administration of oral or parenteral corticosteroids in the 30 days prior to surgery for a chronic condition” were considered to have chronic steroid use. Chemotherapy and radiotherapy for cancer within 90 days prior to surgery were individually captured. Disseminated cancer was considered positive for patients with spread to one or more sites in addition to the primary site and for patients with acute lymphocytic leukemia, acute myelogenous leukemia, and stage IV lymphoma. Renal disease was defined when a patient had a diagnosis of acute or chronic renal failure requiring treatment with peritoneal dialysis, hemodialysis, hemofiltration, or ultrafiltration within 2 weeks prior to surgery. Hepatic disease was considered positive for patients presenting with ascites on physical examination, abdominal ultrasound, or abdominal CT/MR imaging within 30 days prior to operation, or if a patient had esophageal varices documented on by endoscopy or CT scan performed within 6 months prior to surgery, as noted in NSQIP.

An all-inclusive variable, “history indicative of potentially abnormal hemostasis,” was created, indicative of the presence of one or more of the above individual risk factor variables defined above [26]. Sensitivity analyses were conducted using alternate definitions of “history indicative of potentially abnormal hemostasis” to include additional variables: smoking, alcohol consumption, hypertensive medications, weight loss of >10% of body weight in the 6 months preceding surgery. The alternative composite variables did not perform as well as the original composite and were not used (data available upon request).

### Outcomes of interest

Perioperative transfusion is defined as having received at least 1 unit of whole or packed red cells in the peri-operative period, which NISQIP identifies as the patient having been transfused at any time from the start of surgery to 72 hours after surgery. Return to the operating room (OR) is defined as any unplanned return to the OR for a surgical procedure within 30 days of the index operation. Mortality was recoded at 30 days from the time of surgery. Data on readmission was only available for patients that underwent surgery in 2011 and 2012 and was considered positive for patients that had any readmission to the same or another hospital, for any reason, within 30 days of the index surgical procedure.

### General demographics

We merged race categories into Caucasian versus all other. We dichotomized both transfer and functional status, respectively, as admitted from home or transferred from any facility, as well as independent versus dependent functional status. We merged the American Association of Anesthesiologists (ASA) classification into grades 1 and 2 together and 3 and 4 together, as described by the ASA [12]. The presence of residents in the operating room was a surrogate marker for an academic institution or teaching hospital.

### Statistical Analyses

Frequency distributions were used to describe the entire NSQIP surgery patient population and data and cross-tabulation tables were used to compare outcomes across the different predictor values. Pearson's chi-square tests were used to compare differences in outcomes across groups according to number of hemostatic tests undergone and individual predictor variables. Logistic regression was used to model the ability of hemostatic lab tests and patient history to predict the outcomes of interest, and to test the ability of patient history to predict hemostatic lab results. SAS (Version 9.2, SAS Institute) was used for all statistical analyses. A p< 0.05 was considered significant.

## Results

Among the 2,020,533 patients in the ACS-NSQIP database who comprised our global study population (Fig 1; and Methods, above), 872,501 (43.2%) had a PT/INR, 748,568 (37.1%) had an aPTT result, and 1,679,926 (83.1%) had a platelet count; 728,135 (36.0%) had all three tests performed; and 324,638 (16.1%) had none of the three tests done (Table 1). A total of 226,856 patients (11.2%) had a composite history indicative of potentially abnormal hemostasis, with one or more risk factors according to patient history, as defined in the Methods, above. Patients from three specialties comprised five-sixths of the study population, with approximately 62% of the patients in the general surgery group; 11% had an orthopedic procedure, 10% had a vascular procedure and the remainder came from the other six specialties (Table 1). Of the outcomes of interest, the incidence ranged from death in 0.7%, unplanned return to the OR in 3.8%, peri-operative transfusion of blood (RBC) in 5.3%, and re-admission within 30 days of the index surgery in 6.2% of patients (Table 1).

**Table 1:** General demographics, preoperative hemostatic screening tests, patient history variables, and outcomes of interest of all surgery patients (n = 2,020,533).

General demographics	Frequency
Age, years, mean ± SD	56 ± 16
Female	1,176,702 (58.2%)
White	1,445,795 (75.2%)
Admitted from home	1,965,922 (97.3%)
Partially or fully dependent functional status	77,387 (3.8%)
ASA	
1 & 2	1,132,072 (56.2%)
3 & 4	880,715 (43.8%)
5	456 (0.02%)
Prior operation within 30 days	29,762 (2.1%)
Resident in OR	882,482 (58.1%)
Surgical specialty	
General surgery	1,246,455 (61.7%)
Gynecology	100,421 (5.0%)
Neurosurgery	58,305 (2.9%)
Orthopedics	225,509 (11.2%)
Otolaryngology	42,451 (2.1%)
Plastics	39,706 (2.0%)
Thoracic	19,709 (1.0%)
Urology	77,590 (3.8%)
Vascular	210,387 (10.4%)
**Preoperative hemostatic screening tests** †	
INR	872,501 (43.2%)
aPTT	748,568 (37.1%)
Platelet count	1,679,926 (83.1%)
All 3 preoperative screening tests were done	728,135 (36.0%)
No preoperative screening tests	324,638 (16.1%)
**Patient history variables indicative of potential bleeding tendency**	
Bleeding disorder	89,942 (4.5%)
Chronic steroid use	60,252 (3.0%)
Chemotherapy	19,328 (1.0%)
Radiation therapy	11,011 (0.5%)
Disseminated cancer	39,470 (2.0%)
Renal disease	31,175 (1.5%)
Hepatic disease	10,142 (0.5%)
History indicative of potentially abnormal hemostasis‡	226,856 (11.2%)
**Outcomes of interest**	
Perioperative RBC transfusion	107,491 (5.3%)
Return to the OR	75,987 (3.8%)
Mortality	14,827 (0.7%)
Unplanned readmission	50,730 (6.2%)

ASA = American Association of Anesthesiologists; INR = International Normalized Ratio; aPTT = activated partial thromboplastin time; RBC = red blood cell; OR = operating room; SD = standard difference

^†^ Number of patients who underwent each of the preoperative hemostatic tests within 90 days prior to surgery.

^‡^ Patient had one or more of the following risk factors for abnormal hemostasis: history of abnormal bleeding, self-reported family history of bleeding disorders, vitamin K deficiency, currently taking medications that pose a risk for bleeding abnormalities and/or failing to discontinue use of such medications with adequate time for normal hemostasis to be restored, chronic steroid use, chemotherapy and/or radiotherapy for cancer within 90 days prior to surgery, disseminated cancer, renal disease, and/or hepatic disease.

Assessment of outcomes according to the number of preoperative hemostatic tests undergone shows a stepwise association between the number of preoperative screening tests administered and the fraction of suboptimal outcomes. Patients that had all three screening tests (N = 728,135) performed were found to have higher rates of perioperative RBC transfusion, more frequent returns to the OR, an increased incidence of mortality, and a higher 30-day re-admission rate from any cause when compared to patients that had only 1 or 2 tests (N = 967,760), who, in turn, had higher rates than patients with no tests (N = 324,638) done (data not shown). It is not possible in NSQIP, however, to determine or quantify the exact nature of this relationship nor to identify whether this relationship could be identified only by screening laboratory tests in asymptomatic patients, the features in NSQIP used for the history screen, or other variables. Nonetheless, in each category of test, 88.6 to 91.2% of patients had results in the normal range, and most abnormalities were mild (range 8.9–11.4% for any abnormality, mild to severe or low or elevated); severely abnormal results were not common, and ranged from 0.3% of PT/INR results to 1.3% of aPTT results (Table 2). In addition, when examining Tables 1 or 2, one sees that while essentially all patients who had either an aPTT or a PT/INR level also had a platelet count performed, the reverse is not true. In general, as tests of coagulation or the platelet count became more abnormal, the incidence of perioperative complications increased as well, although the specificity of these findings (range, 0.89 to 0.94) are more robust than the sensitivity (range, 0.08–0.31; see Table 2).

**Table 2:** Outcomes stratified by INR values, aPTT values, and platelet count in all surgery patients (N = 2,020,533).

Test and result	No. of patients (%)	No. (%)			
		Perioperative RBC transfusion	Return to the OR	Mortality	Unplanned readmission
**INR**	**872,501**				
Normal	793,277 (90.9%)	69,609 (8.8%)	36,224 (4.6%)	8,135 (1.0%)	26,166 (7.9%)
Mildly abnormal	76,678 (8.8%)	12,126 (5.8%)	7,495 (9.8%)	3,572 (4.7%)	3,769 (13.8%)
Severely abnormal INR	2,546 (0.3%)	283 (11.1%)	251 (9.9%)	65 (2.6%)	134 (14.1%)
All abnormal	79,224 (9.1%)	12,409 (15.7%)	7,746 (9.8%)	3,637 (4.6%)	12,409 (15.7%)
P-value*		**<0.001**	**<0.001**	**<0.001**	**<0.001**
Sensitivity		0.15	0.18	0.31	0.15
Specificity		0.92	0.91	0.91	0.92
**aPTT**	**748,568**				
Normal	663,274 (88.6%)	57,884 (8.7%)	30,321 (4.6%)	7,395 (1.1%)	20,783 (7.8%)
Mildly abnormal	75,491 (10.1%)	11,231 (14.9%)	6,852 (9.1%)	2,392 (3.2%)	3,256 (11.9%)
Severely abnormal	9,803 (1.3%)	1,746 (17.8%)	1,431 (14.6%)	377 (3.9%)	563 (16.7%)
All abnormal	85,294 (11.4%)	12,977 (15.2%)	8283 (9.7%)	2,769 (3.3%)	3819 (12.4%)
P-value*		**<0.001**	**<0.001**	**<0.001**	**<0.001**
Sensitivity		0.18	0.21	0.27	0.16
Specificity		0.89	0.89	0.89	0.92
**Platelet count**	**1,679,926**				
Normal	1,532,027 (91.2%)	85,633 (5.6%)	58,844 (3.8%)	11,024 (0.7%)	40846 (6.5%)
Abnormal low	105,115 (6.3%)	11,914 (11.3%)	5,605 (5.3%)	2,559 (2.4%)	4372 (9.5%)
Abnormal high	42,784 (2.6%)	6,427 (15.0%)	3,715 (8.7%)	813 (1.9%)	1572 (11.5%)
P-value†		**<0.001**	**<0.001**	**<0.001**	**<0.001**
Sensitivity‡		0.11	0.08	0.18	0.09
Sensitivity‡		0.94	0.94	0.94	0.94

aPTT = activated partial thromboplastin time; INR = International Normalized Ratio; RBC = red blood cell; OR = operating room

* All abnormal compared with normal.

^†^ Abnormal low platelet count compared with normal platelet count.

^‡^ Sensitivity and specificity are for abnormal low platelet count only.

§ Odd ratios and p values that are significant are bolded.

In patients who had all three tests (N = 728,135), the number of abnormal preoperative hemostatic tests was significantly associated with each adverse outcome (Table 3). Patients who had one abnormal test result were more likely to have had each adverse outcome compared to patients with three normal test results, and patients with two or three abnormal test results had the highest odds for poor outcomes (Table 3).

**Table 3:** Outcome Odds ratios by number of abnormal hemostasis test results in 728,135 surgery patients who underwent all 3 hemostasis tests.

Outcome Variables	No. of patients	All 3 tests are within normal range (n = 554,551)	One abnormal test (n = 129,641)	Odds Ratio* (95% CI)	Two or three abnormal tests (n = 43,943)	Odds Ratio (95% CI)*	Global P-Value†
Perioperative RBC transfusion	69,729	43,389 (7.8%)	17,975 (13.9%)	**1.9 (1.86–1.93)**	8,365 (19.0%)	**2.8 (2.7–2.8)**	**<0.001**
Return to the OR	37,687	22,982 (4.1%)	9,595 (7.4%)	**1.8 (1.8–1.9)**	5,110 (11.6%)	**3.0 (2.9–3.1)**	**<0.001**
Mortality	10,013	4,579 (0.8%)	3,114 (2.4%)	**3.0 (2.8–3.1)**	2,320 (5.3%)	**6.7 (6.4–7.0)**	**<0.001**
Unplanned readmission	24,136	16,539 (7.3%)	5,395 (11.1%)	**1.6 (1.5–1.6)**	2,202 (14.7%)	**2.2 (2.1–2.3)**	**<0.001**

CI = confidence interval; OR = operating room; RBC = red blood cell

* Odd ratios are relative to all three tests within normal range.

^†^ Pearson's chi-square test used to compare differences in outcomes across all groups.

‡ Odd ratios and p values that are significant are bolded.

Next, we evaluated the relationship between our composite history indicative of potentially abnormal hemostasis (“positive history”) and each adverse outcome of interest in the full study sample of 2,020,533 patients (Table 4). Compared to those without a history (“negative history”), patients with a “positive history” had significantly higher odds for each adverse outcome. In fact, these odds were greater than those seen with one abnormal test out of three and equivalent to those patients who had two or three abnormal tests (Table 4); in all cases, the results are significant (p<0.001). Furthermore, while the specificity of the positive history composite is essentially equivalent to the results of one or more abnormal lab tests (range, 0.89–0.90), the sensitivity is somewhat more robust (range, 0.23–0.45).

**Table 4:** Outcome Odds Ratios by patient “history indicative of potentially abnormal hemostasis” in all surgery patients (N = 2,020,533).

Outcome Variables	No. of patients	No history* (n = 1,793,677)	History* (n = 226,856)	Odds Ratio (95% CI)	P-Value	Sensitivity	Specificity
Perioperative RBC transfusion	107,491	79,403 (4.4%)	28,088 (12.4%)	**3.1 (3.0–3.1)**	**<0.001**	0.26	0.90
Return to the OR	75,987	58,563 (3.3%)	17,424 (7.7%)	**2.5 (2.4–2.5)**	**<0.001**	0.23	0.89
Mortality	14,827	8,168 (0.05%)	6,659 (2.9%)	**6.6 (6.4–6.8)**	**<0.001**	0.45	0.89
Unplanned readmission	50,730	39,310 (5.4%)	11,420 (13.0%)	**2.6 (2.6–2.7)**	**<0.001**	0.23	0.91

CI = confidence interval; RBC = red blood cell; OR = operating room

* History = History indicative of potentially abnormal hemostasis

† Odd ratios and p values that are significant are bolded.

To examine the relationship between history and abnormal tests, we limited our sample size to the 728,135 patients that underwent all 3 tests (Table 5). We found that presence of history was significantly associated with each type of abnormal test finding, so as one might expect there is a clear and strong association between patient hemostasis history and abnormal test results.

**Table 5:** Abnormal screening test Odds Ratios by patient “history indicative of potentially abnormal hemostasis” in surgery patients screened with all 3 hemostasis tests (n = 728,135).

Test Findings	No. of patients	No history* (N = 1,286)	History* (N = 1,286)	Odds Ratio (95% CI)	P-Value
Mildly abnormal INR	59,342	33,906	25,436	**4.1 (4.0–4.2)**	**<0.001**
Severely abnormal INR	1,749	996	753	**3.5 (3.2–3.9)**	**<0.001**
All abnormal INR	61,091	34,902	26,189	**4.1 (4.0–4.2)**	**<0.001**
Mildly abnormal aPTT	73,560	49,233	24,327	**2.6 (2.5–2.6)**	**<0.001**
Severely abnormal aPTT	9,359	4,460	4,899	**5.2 (5.0–5.5)**	**<0.001**
All abnormal aPTT	82,919	53,693	29,226	**3.0 (2.9–3.0)**	**<0.001**
Abnormal low platelet count	57,050	36,702	20,348	**2.9 (2.8–2.9)**	**<0.001**
Abnormal high platelet count	23,660	17,323	6,337	**1.7 (1.7–1.8)**	**<0.001**

aPTT = activated partial thromboplastin time; CI = confidence interval; INR = International Normalized Ratio; OR = operating room; RBC = red blood cell

* History = History indicative of potentially abnormal hemostasis

† Odd ratios and p values that are significant are bolded.

We then assessed the predictive value of a “history indicative of potentially abnormal hemostasis” compared to hemostatic screening, based on the percentage of each of the outcomes of interest that were detected in patients that were screened with all three tests (Table 6). The percentage of patients captured with each of the poor outcomes was approximately the same when using a “positive history” alone compared with the presence of one or more screening test(s) being abnormal alone. Even the combination of a “positive history” with one or more abnormal test results detected only between 46 and 71% of patients with any one of the outcomes of interest. The remainder of the patients—roughly one-third to one-half in each instance, whether it was a blood transfusion, unexpected return to the OR, re-admission to the hospital within 30 days, or mortality—was not identified by either abnormal history or preoperative hemostatic screening.

**Table 6:** Predictive value of “patient history indicating potentially abnormal coagulation”, abnormal hemostatic test results, both, and neither in patients screened with all 3 hemostatic tests (n = 728,135).

Outcome Variables	No. of patients	History*	≥1 abnormal test	With history* and/or ≥1 abnormal test	Without history* and no abnormal coagulation tests
No. of patients		129,353	173,584	245,273	482,862
Perioperative RBC transfusion	69,729	29.0%	37.8%	51.2%	48.8%
Return to the OR	37,687	30.9%	39.0%	52.7%	47.3%
Mortality	10,013	48.4%	54.3%	70.8%	29.2%
Unplanned readmission	47,446	28.1%	31.5%	46.2%	53.8%

* History = History indicative of potentially abnormal hemostasis

Since roughly 61% of our sample is comprised of general surgery patients, the findings in this specialty may influence the overall results. To assess the generalizability of our findings to other surgical specialties, we chose to conduct sensitivity analyses according to the disciplines outlined in Table 1 (see S1–S9 Tables). We chose to limit our subsample analyses to include the top ten surgeries performed for each subspecialty for the ten most prevalent diagnoses, to assess those procedures that address the most common conditions found in the vast majority of surgical practices. In general, we found similar results in each of the surgical subspecialties; detailed results by specialty are reviewed in the supplemental text (S1 Text) and tables (S1–S9 Tables).

## Discussion

### Overview

In a large sample of 2,020,533 people, over a recent 7-year period, who underwent elective, non-cardiac surgery, across a range of specialties, roughly one-third (36%) had all three pre-operative hemostatic screening tests. In the last test performed immediately prior to the index surgery, about 40% had a PT or an aPTT and 83% had a platelet count and most of those with a PT had an aPTT (Table 1); we do not know whether this test was the only test of that type performed or if there were abnormalities in the test(s) performed prior to this index lab result, only that this was the test that preceded the operation. Similar results were found in each subspecialty, although there is more variability in practice seen (see S1 Text). Mild abnormalities of both the PT and aPTT occurred in 8.8–10.1% of patients but severe abnormalities were rare (0.3–1.3%); alterations in platelet counts, both thrombocytopenia and elevated platelet levels, were more common, identified in up to 12% of the patients (Table 2). In general, the percentage of patients with any of the four outcomes of interest was higher in those with abnormalities of 1, 2, or 3 of the tests compared to those with normal values. Most patients with 1 or more abnormal test, as well as those with normal results, had no peri-operative complication (Tables 2 and 4). However, patients with a “history indicative of potentially abnormal hemostasis” (“positive history”) had results nearly identical to those with laboratory abnormalities (Table 5); and, a “positive history” increased significantly the odds of any laboratory test being abnormal from 1.7–5.2-fold compared to “negative history” patients (Table 5). Finally, the predictive value of a “positive history,” compared with hemostatic screening was assessed by determining the percentage of each of the outcomes of interest detected in patients who were screened using all three tests (Table 6). The percentage captured with each suboptimal outcome of interest was roughly the same when using “positive history” compared with one or more abnormal test (remembering that nearly all patients with a PT had an aPTT but not all patients with a platelet count had a coagulation study). Combining “positive history” and abnormal test results as a composite (one, the other, or both present) identified only about half of patients who received blood, had an unplanned return to surgery, or were re-admitted after surgery and discharge, although just over 70% of deaths were detected. The remaining patients with one or more significant peri-operative complication were not captured by either hemostatic screening tests or features of the composite history.

### Clinical Implications

Our findings support other work that has suggested a limited benefit from routine or standing pre-operative hemostatic testing. Our data, both from the entire sample, as well as from examination of the most common procedures done in each of the nine non-cardiac specialties reported in NSQIP, suggest that surgeons and other clinicians were somewhat selective as to which patients had preoperative laboratory testing of hemostasis, with a positive association between number of tests ordered and each outcome of interest. However, the vast majority of patients who had testing—as well as the greater proportion of those who did not have testing—had no clinical risk factors for abnormal testing, had results in the normal range, and did not have one of the adverse outcomes of interest. If one were to limit testing to those with a history suggestive or indicative of abnormal hemostasis (roughly 11.2% of this population), and were to assume no additional testing or diagnostic studies of any kind were needed, with an average cost of US$21.00, 31.00, and $34.00 for each of a PT/INR, aPTT, and platelet count (sum $86.00; source, the Healthcare Bluebook, https://www.healthcarebluebook. com; accessed 3/2/2015), then the savings in this cohort would be as much as $154.3 million yearly (0.888 of 2,020,533, or 1,794,233 with no history multiplied by $86.00); and, if these figures were used for all 15 million operating room in the US each year, a savings of $1.15 billion per year (0.888 times 15 million equals 13,320,000 patients, multiplied by $86.00) [1–6].

These data illustrate, in a large population, across a range of surgical specialties and procedures, that hemostatic testing is widespread yet inconstant across the broad spectrum of American hospitals and surgical practices in NSQIP and does not appear to be guided strictly by variables derived from the patient’s pre-operative history and physical examination. Hemostatic test accuracy was suboptimal, with low sensitivity to predict several clinically-relevant outcomes of interest, including perioperative transfusion, unplanned return to the operating room for a surgical procedure within 30 days of the index operation, readmission to a hospital and death within 30 days of surgery. Using a history composite indicative of potentially abnormal hemostasis—a composite that contains simple elements that can be obtained from essentially every patient who is to undergo elective but major, non-cardiac surgery—as a tool to guide selection of patients for preoperative screening, is a medically-reasonable and cost-effective strategy.

## Limitations

We recognize limitations in this study. The NSQIP database contains only those who underwent surgery, so patients in whom persistent hemostatic abnormalities that preclude surgery would not be included, nor would patients in whom a clinically-relevant abnormality was identified and fully resolved be identified. However, previous work suggests that few patients have surgery cancelled for this reason and the data reported here, across a broad spectrum of surgical specialties suggests that significant abnormalities are ultimately not common [7, 20–22, 24–26, 32–35]. Patients who required emergency surgery, where coagulation abnormalities due to trauma, disease, or medication, and which may not be easily controlled, were excluded, as were patients undergoing cardiac surgery, where hemostatic and/or coagulation disorders are more common as a result of cardiac bypass and where blood transfusion is frequent; the results we report here should not be extended to these populations. The NSQIP database only records the last laboratory value(s) performed before surgery, up to 90 days before that index surgery, so if multiple rounds of testing had been done, or an intervention done to reverse or correct a hemostatic abnormality, prior to surgery, this is not recorded in NSQIP. A study by Ng et al, in patients undergoing non-cardiac surgery, revealed that those with abnormal hemostasis more commonly received fresh-frozen plasma or platelet transfusions, as part of a pre-operative change in the plan of care, than were whose hemostatic tests were normal; however, outcomes in terms of blood loss, bleeding complications, and mortality were no different^30^.

We recognize that none of our outcomes of interest is solely dependent upon hemostasis; that the patient sample sets in NSQIP may not represent faithfully the entire population undergoing surgery in the nine, non-cardiac sub-specialties we examined; that other variables that influence peri-operative outcomes may not be captured in NSQIP; and, finally, that this is a retrospective analysis of prospectively-collected data that was not designed specifically to interrogate these populations on the basis of history or laboratory testing alone, while controlling all other variables. However, NSQIP has been shown to be representative of the US population in terms of sex and race and is drawn from a large number of hospitals (larger and smaller facilities, teaching and non-teaching hospitals) that represent the spectrum of clinical practice in the US, and provides a large and diverse sample size with robust pre-, intra-, and post-operative data on patients who undergo surgery, followed by an in-patient stay, with numerous outcomes of interest for the entire healthcare community (available at: www.acsnisquip.org/default.jsp) [14–16, 35]. Data in NSQIP are collected in a standardized fashion at every site, with strict definition of the variables; annual quality checks demonstrate accurate, precise, and reproducible data collection, with >95% 30-day outcome follow-up, in all institutions, across consecutive collection cycles [14–16,35]. NSQIP has been demonstrated in numerous studies to provide an effective database for the analysis of surgical quality and outcomes and becomes more reliable as the sample sizes increase as well as being strong in terms of prediction of major events such as death, unplanned return to the OR or re-admission, outcomes that were the main foci of this investigation [14–16, 35].

## Conclusions

This study is the first to measure the rate of preoperative testing in a very large subset (>2 million subjects, from 2006 to 2012) of the population of US patients undergoing common types of major, elective, non-cardiac surgery, followed by hospitalization, who were evaluated prospectively, with verified 30-day outcomes; as well as the first to assess the utility of common laboratory screening, compared with the use of simple features of a patient’s history that might indicate potentially abnormal hemostasis, across several, clinically-relevant operative outcomes, both across the larger population studied as well as in the most common surgical procedures performed in nine, non-cardiac specialties. Preoperative hemostasis testing is widespread in the population, with about 84% having at least one of these tests, and 36% having all three even though a history suggestive of potentially abnormal hemostasis was present in only 11% of patients. An abnormal INR, aPTT, and a low platelet count were each associated with a peri-operative blood transfusion, unplanned return to the OR, death, or unplanned re-admission, with a large, indiscriminant overlap with those who did not have an abnormality and a negative outcome of interest; low sensitivity was common to all three tests, which suggests that they are poor screening tool(s) in for most patients. A “positive history” was similarly significantly associated with each and all of the negative outcomes of interest and was at least as predictive as one (or more) abnormal test result. The combination of a “positive history” and one or more abnormal test identified only 70% of the patients who died and 46–53% of patients who had one of the other three outcomes of interest, which indicates that other factors play a role in outcomes, including additional patient factors; operative team dynamics; surgeon selection and decision-making, experience, and skill; in addition to other features. Excess costs associated with screening tests, combined with the limited predictive value of each test, across a broad spectrum of elective surgeries, suggests that limiting hemostasis testing to a more select group of patients presenting with a history indicative of abnormal hemostasis may be reasonable, efficient, efficacious, and cost-effective.

## Supporting Information

S1 Table general surgery patients. Table S1A. General demographics, preoperative hemostatic screening tests, patient history variables, and outcomes of interest of general surgery patients (n = 289,982). Table S1B. Outcomes stratified by INR values, aPTT values, and platelet count in all general surgery patients (n = 289,982). Table S1C. Outcome odds ratios by number of abnormal hemostasis test results in 65,784 general surgery patients who underwent all 3 hemostasis tests. Table S1D. Outcome odds ratios by patient “history indicative of potentially abnormal hemostasis” in all general surgery patients (n = 289,982). Table S1E. Abnormal screening test odds ratios by patient “history indicative of potentially abnormal hemostasis” in general surgery patients screened with all 3 hemostasis tests (n = 65,784). Table S1F. Predictive value of “patient history indicating potentially abnormal coagulation”, abnormal hemostatic test results, both, and neither in general patients screened with all 3 hemostatic tests (n = 65,784).
(DOCX)

S2 Table gynecology surgery patients. Table S2A. General demographics, preoperative hemostatic screening tests, patient history variables, and outcomes of interest of gynecology surgery patients (n = 33,235). Table S2B. General demographics, preoperative hemostatic screening tests, patient history variables, and outcomes of interest of gynecology surgery patients (n = 33,235). Table S2C. Outcome odds ratios by number of abnormal hemostasis test results in 6,814 gynecology surgery patients who underwent all 3 hemostasis tests. Table S2D. Outcome odds ratios by patient “history indicative of potentially abnormal hemostasis” in all gynecology surgery patients (n = 33,235). Table S2E. Abnormal screening test odds ratios by patient “history indicative of potentially abnormal hemostasis” in gynecology surgery patients screened with all 3 hemostasis tests (n = 6,814). Table S2F. Predictive value of “patient history indicating potentially abnormal coagulation”, abnormal hemostatic test results, both, and neither in gynecology patients screened with all 3 hemostatic tests (n = 6,814).
(DOCX)

S3 Table neurosurgery patients. Table S3A. General demographics, preoperative hemostatic screening tests, patient history variables, and outcomes of interest of neurosurgery patients (n = 24,453). Table S3B. Outcomes stratified by INR values, aPTT values, and platelet count in all neurosurgery patients (n = 24,453). Table S3C. Outcome odds ratios by number of abnormal hemostasis test results in 14,500 neurosurgery patients who underwent all 3 hemostasis tests. Table S3D. Outcome odds ratios by patient “history indicative of potentially abnormal hemostasis” in all neurosurgery patients (n = 24,453). Table S3E. Abnormal screening test odds ratios by patient “history indicative of potentially abnormal hemostasis” in neurosurgery patients screened with all 3 hemostasis tests (n = 14,500). Table S3F. Predictive value of “patient history indicating potentially abnormal coagulation”, abnormal hemostatic test results, both, and neither in neurosurgery patients screened with all 3 hemostatic tests (n = 14,500).
(DOCX)

S4 Table orthopedic surgery patients. Table S4A. General demographics, preoperative hemostatic screening tests, patient history variables, and outcomes of interest of orthopedic surgery patients (n = 90,627). Table S4B. Outcomes stratified by INR values, aPTT values, and platelet count in all orthopedic surgery patients (n = 90,627). Table S4C. Outcome odds ratios by number of abnormal hemostasis test results in 41,445 orthopedic surgery patients who underwent all 3 hemostasis tests.Table S4D. Outcome odds ratios by patient “history indicative of potentially abnormal hemostasis” in all orthopedic surgery patients (n = 90,627). Table S4E. Abnormal screening test odds ratios by patient “history indicative of potentially abnormal hemostasis” in orthopedic surgery patients screened with all 3 hemostasis tests (n = 41,445). Table S4F. Predictive value of “patient history indicating potentially abnormal coagulation”, abnormal hemostatic test results, both, and neither in orthopedic surgery patients screened with all 3 hemostatic tests (n = 41,445).
(DOCX)

S5 Table otolaryngology patients. Table S5A. General demographics, preoperative hemostatic screening tests, patient history variables, and outcomes of interest of otolaryngology patients (n = 14,706). Table S5B. Outcomes stratified by INR values, aPTT values, and platelet count in all otolaryngology surgery patients (n = 14,706). Table S5C. Outcome odds ratios by number of abnormal hemostasis test results in 4,006 otolaryngology surgery patients who underwent all 3 hemostasis tests. Table S5D. Outcome odds ratios by patient “history indicative of potentially abnormal hemostasis” in all otolaryngology patients (n = 14,706). Table S5E. Abnormal screening test odds ratios by patient “history indicative of potentially abnormal hemostasis” in otolaryngology patients screened with all 3 hemostasis tests (n = 4,006). Table S5F. Predictive value of “patient history indicating potentially abnormal coagulation”, abnormal hemostatic test results, both, and neither in otolaryngology patients screened with all 3 hemostatic tests (n = 4,006).
(DOCX)

S6 Table plastic surgery patients. Table S6A. General demographics, preoperative hemostatic screening tests, patient history variables, and outcomes of interest of plastic surgery patients (n = 15,399). Table S6B. Outcomes stratified by INR values, aPTT values, and platelet count in all plastic surgery patients (n = 15,399). Table S6C. Outcome odds ratios by number of abnormal hemostasis test results in 2,892 plastic surgery patients who underwent all 3 hemostasis tests. Table S6D. Outcome odds ratios by patient “history indicative of potentially abnormal hemostasis” in all plastic surgery patients (n = 15,399). Table S6E. Abnormal screening test odds ratios by patient “history indicative of potentially abnormal hemostasis” in plastic surgery patients screened with all 3 hemostasis tests (n = 2,892). Table S6F. Predictive value of “patient history indicating potentially abnormal coagulation”, abnormal hemostatic test results, both, and neither in plastic surgery patients screened with all 3 hemostatic tests (n = 2,892).
(DOCX)

S7 Table thoracic surgery patients. Table S7A. General demographics, preoperative hemostatic screening tests, patient history variables, and outcomes of interest of thoracic surgery patients (n = 7,758). Table S7B. Outcomes stratified by INR values, aPTT values, and platelet count in all thoracic surgery patients (n = 7,758). Table S7C. Outcome odds ratios by number of abnormal hemostasis test results in 5,139 thoracic surgery patients who underwent all 3 hemostasis tests. Table S7D. Outcome odds ratios by number of abnormal hemostasis test results in 5,139 thoracic surgery patients who underwent all 3 hemostasis tests. Table S7E. Abnormal screening test odds ratios by patient “history indicative of potentially abnormal hemostasis” in thoracic surgery patients screened with all 3 hemostasis tests (n = 5,139). Table S7F. Predictive values, stratified by the presence or absence of a positive history for potentially abnormal hemostasis, and pre-operative screening in all patients (n = 728,135) who underwent all 3 hematologic tests prior to surgery.
(DOCX)

S8 Table urological surgery patients. Table S8A. General demographics, preoperative hemostatic screening tests, patient history variables, and outcomes of interest of urological surgery patients (n = 34,258). Table S8B. Outcomes stratified by INR values, aPTT values, and platelet count in all urological surgery patients (n = 34,258). Table S8C. Outcome odds ratios by number of abnormal hemostasis test results in 14,254 urological surgery patients who underwent all 3 hemostasis tests. Table S9D. Outcome odds ratios by patient “history indicative of potentially abnormal hemostasis” in all urological surgery patients (n = 34,258). Table S8E. Abnormal screening test odds ratios by patient “history indicative of potentially abnormal hemostasis” in urological surgery patients screened with all 3 hemostasis tests (n = 14,254). Table S8F. Predictive value of “patient history indicating potentially abnormal coagulation”, abnormal hemostatic test results, both, and neither in urological surgery patients screened with all 3 hemostatic tests (n = 14,254).
(DOCX)

S9 Table vascular surgery patients. Table S9A. General demographics, preoperative hemostatic screening tests, patient history variables, and outcomes of interest of vascular surgery patients (n = 83,101). Table S9B. Outcomes stratified by INR values, aPTT values, and platelet count in all vascular surgery patients (n = 83,101). Table S9C. Outcome odds ratios by number of abnormal hemostasis test results in 50,839 vascular surgery patients who underwent all 3 hemostasis tests. Table S9D. Outcome odds ratios by patient “history indicative of potentially abnormal hemostasis” in all vascular surgery patients (n = 83,101). Table S9E. Abnormal screening test odds ratios by patient “history indicative of potentially abnormal hemostasis” in vascular surgery patients screened with all 3 hemostasis tests (n = 50,839). Table S9F. Predictive value of “patient history indicating potentially abnormal coagulation”, abnormal hemostatic test results, both, and neither in vascular surgery patients screened with all 3 hemostatic tests (n = 50,839).
(DOCX)

S1 Text (DOCX)

## Abbreviations

PT: prothrombin time
INR: international normalized ration
aPTT: activated partial thromboplastin time
OR: operating room
CT: computed tomography
MR: magnetic resonance
