# Supplementary material for: Use and Utility of Hemostatic Screening in Adults Undergoing Elective, Non-Cardiac Surgery
Source: PLoS One. 2015 Dec 1;10(12):e0139139. doi: 10.1371/journal.pone.0139139 (PMC4666643; doi:10.1371/journal.pone.0139139)
Supplement: S1 Table — Table S1A. General demographics, preoperative hemostatic screening tests, patient history variables, and outcomes of interest of general surgery patients (n = 289,982). Table S1B. Outcomes stratified by INR values, aPTT values, and platelet count in all general surgery patients (n = 289,982). Table S1C. Outcome odds ratios by number of abnormal hemostasis test results in 65,784 general surgery patients who underwent all 3 hemostasis tests. Table S1D. Outcome odds ratios by patient “history indicative of potentially abnormal hemostasis” in all general surgery patients (n = 289,982). Table S1E. Abnormal screening test odds ratios by patient “history indicative of potentially abnormal hemostasis” in general surgery patients screened with all 3 hemostasis tests (n = 65,784). Table S1F. Predictive value of “patient history indicating potentially abnormal coagulation”, abnormal hemostatic test results, both, and neither in general patients screened with all 3 hemostatic tests (n = 65,784). (DOCX) [file pone.0139139.s001.docx]

**Table S1A: General demographics, preoperative hemostatic screening tests, patient history variables, and outcomes of interest of general surgery patients** (n=289,982)

| **General demographics** | **Frequency** |
| --- | --- |
| Age, years, mean ± SD | 52 ± 16 |
| Female | 175,518 (60.5%) |
| White | 203,924 (73.5%) |
| Admitted from home | 287,911 (99.3%) |
| Partially or fully dependent functional status | 2,605 (0.9%) |
| ASA | |
| 1 & 2 | 188,984 (65.3%) |
| 3 & 4 | 100,411 (34.7%) |
| 5 | 20 (0.01%) |
| Prior operation within 30 days | 2,261 (1.0%) |
| Resident in OR | 136,893 (59.4%) |
| **Preoperative hemostatic screening tests†** | |
| INR | 79,137 (27.3%) |
| aPTT | 68,464 (23.6%) |
| Platelet count | 221,593 (76.4%) |
| All 3 preoperative screening tests were done | 65,784 (22.7%) |
| No preoperative screening tests | 66,066 (22.8%) |
| **Patient history variables indicative of potential bleeding tendency** | |
| Bleeding disorder | 5,528 (1.9%) |
| Chronic steroid use | 4,717 (1.6%) |
| Chemotherapy | 1,250 (0.4%) |
| Radiation therapy | 212 (0.1%) |
| Disseminated cancer | 891 (0.3%) |
| Renal disease | 1,381 (0.5%) |
| Hepatic disease | 796 (0.3%) |
| History indicative of potentially abnormal hemostasis‡ | 13,558 (4.7%) |
| **Outcomes of interest** | |
| Perioperative RBC transfusion | 1,278 (0.4%) |
| Return to the OR | 6,887 (2.4%) |
| Mortality | 340 (0.1%) |
| Unplanned readmission | 3,999 (1.4%) |

Definitions: SD, standard deviation or standard difference; ASA = American Association of Anesthesiologists; OR, operating room; INR = International Normalized Ratio; aPTT = activated partial thromboplastin time; RBC = red blood cell;

*Procedures performed, by CPT codes, included, in descending order of frequency, are: 47562, 49505, 43644, 49560, 47563, 49585, 19301, 19303, 19125, 44204.

**Diagnoses included (ICD-9 code), in descending order of frequency, are: 278.0, 550.90, 574.10, 553.21, 174.9, 553.1, 562.11, 233.0, 574.20, 540.9.

† Number of patients who underwent each of the preoperative hemostatic tests within 90 days prior to surgery.

‡ Patient had one or more of the following risk factors for abnormal haemostasis: history of abnormal bleeding, self-reported family history of bleeding disorders, vitamin K deficiency, currently taking medications that pose a risk for bleeding abnormalities and/or failing to discontinue use of such medications with adequate time for normal hemostasis to be restored, chronic steroid use, chemotherapy and/or radiotherapy for cancer within 90 days prior to surgery, disseminated cancer, renal disease, and/or hepatic disease.

**Table S1B: Outcomes stratified by INR values, aPTT values, and platelet count in all general surgery patients** (n=289,982)

| Test and result | No. of patients (%) | No. (%) | | | |
| --- | --- | --- | --- | --- | --- |
|  |  | Perioperative RBC transfusion | Return to the OR | Mortality | Unplanned readmission |
| INR | 79,137 |  |  |  |  |
| Normal | 74,024 (93.5%) | 503 (0.7%) | 1,778 (2.4%) | 122 (0.2%) | 1,373 (5.6%) |
| Mildly abnormal | 4,919 (6.2%) | 81 (1.7%) | 180 (3.7%) | 54 (1.1%) | 175 (11.2%) |
| Severely abnormal INR | 194 (0.3%) | 2 (1.0%) | 6 (3.1%) | 0 (0.0%) | 8 (12.9%) |
| All abnormal | 5,113 (6.5%) | 83 (1.6%) | 186 (3.6%) | 54 (1.1%) | 183 (11.3%) |
| P-value* |  | **<0.001** | **<0.001** | **<0.001** | **<0.001** |
| Sensitivity |  | 0.15 | 0.09 | 0.31 | 0.12 |
| Specificity |  | 094 | 0.94 | 0.94 | 0.94 |
| aPTT | 68,464 |  |  |  |  |
| Normal | 63,182 (92.3%) | 414 (0.7%) | 1,489 (2.4%) | 123 (0.2%) | 4,147 (5.8%) |
| Mildly abnormal | 5,005 (7.3%) | 82 (1.7%) | 148 (3.0%) | 25 (0.5%) | 122 (7.7%) |
| Severely abnormal | 277 (0.4%) | 3 (1.1%) | 9 (3.3%) | 2 (0.7%) | 10 (13.9%) |
| All abnormal | 5,282 (7.7%) | 85 (1.6%) | 157 (3.0%) | 27 (0.5%) | 132 (7.9%) |
| P-value* |  | **<0.001** | **0.01** | **<0.001** | **<0.001** |
| Sensitivity |  | 0.17 | 0.10 | 0.18 | 0.10 |
| Specificity |  | 0.92 | 0.92 | 0.92 | 0.92 |
| Platelet count | 221,593 |  |  |  |  |
| Normal | 207,878 (93.8%) | 1,044 (0.5%) | 5,111 (2.5%) | 247 (0.1%) | 3,186 (4.5%) |
| Abnormal low | 10,772 (4.9%) | 76 (0.7%) | 255 (2.4%) | 47 (0.4%) | 245 (5.8%) |
| Abnormal high | 2,943 (1.3%) | 26 (0.9%) | 100 (3.4%) | 4 (0.1%) | 38 (5.2%) |
| P-value† |  | **0.001** | **<0.01** | **<0.001** | **<0.001** |
| Sensitivity‡ |  | 0.07 | 0.05 | 0.16 | 0.07 |
| Sensitivity‡ |  | 0.95 | 0.95 | 0.95 | 0.94 |

Definitions: No, number; aPTT = activated partial thromboplastin time; INR = International Normalized Ratio; RBC = red blood cell; OR = operating room

* All abnormal compared with normal. † Abnormal low platelet count compared with normal platelet count.

‡ Sensitivity and specificity are for abnormal low platelet count only. § Odd ratios and p values that are significant are bolded.

**Table S1C: Outcome odds ratios by number of abnormal hemostasis test results in 65,784 general surgery patients who underwent all 3 hemostasis tests**

| Outcome Variables | No. of patients | All 3 tests are within normal range  (n=54,930) | One abnormal test  (n=8,595) | Odds Ratio* (95% CI) | Two or three abnormal tests  (n=2,259) | Odds Ratio (95% CI)* | Global P-Value† |
| --- | --- | --- | --- | --- | --- | --- | --- |
| Perioperative RBC transfusion | 481 | 51 (2.3%) | 95 (1.1%) | **1.8 (1.4-2.3)** | 335 (0.6%) | **3.8 (2.8-5.1)** | **<0.001** |
| Return to the OR | 1,584 | 1,275 (2.3%) | 230 (2.1%) | 1.1 (1.0–1.3) | 79 (3.5%) | **1.5 (1.2-1.9)** | **<0.001** |
| Mortality | 146 | 88 (0.2%) | 31 (0.4%) | **2.3 (1.5-3.4)** | 27 (1.2%) | **7.5 (4.9-11.6)** | **<0.001** |
| Unplanned readmission | 1,243 | 977 (5.6%) | 184 (6.8%) | 1.2 (1.0-1.4) | 82 (11.6%) | **2.2 (1.7-2.8)** | **<0.001** |

Definitions: No, number; CI = confidence interval; OR = operating room; RBC = red blood cell

* Odd ratios are relative to all three tests within normal range.

† Pearson's chi-square test used to compare differences in outcomes across all groups.

‡ Odd ratios and p values that are significant are bolded.

**Table S1D: Outcome odds ratios by patient “history indicative of potentially abnormal hemostasis” in all general surgery patients** (n=289,982)

| Outcome Variables | No. of patients | No history*  (n=276,424) | History*  (n=13,558) | Odds Ratio  (95% CI) | P-Value | Sensitivity | Specificity |
| --- | --- | --- | --- | --- | --- | --- | --- |
| Perioperative RBC transfusion | 1,278 | 1,113 (0.5%) | 165 (1.2%) | **3.0 (2.6-3.6)** | **<0.001** | 0.13 | 0.96 |
| Return to the OR | 6,887 | 6,105 (2.3%) | 482 (3.6%) | **1.6 (1.4-1.7)** | **<0.001** | 0.07 | 0.95 |
| Mortality | 340 | 258 (0.1%) | 82 (0.6%) | **6.5 (5.1-8.4)** | **<0.001** | 0.24 | 0.95 |
| Unplanned readmission | 3,999 | 3587 (3.8%) | 412 (8.6%) | **2.4 (2.1-2.6)** | **<0.001** | 0.10 | 0.95 |

Definitions: No, number; CI = confidence interval; RBC = red blood cell; OR = operating room

* History = History indicative of potentially abnormal hemostasis

† Odd ratios and p values that are significant are bolded.

**Table S1E: Abnormal screening test odds ratios by patient “history indicative of potentially abnormal hemostasis” in general surgery patients screened with all 3 hemostasis tests** (n=65,784)

| Test Findings | No. of patients | No history*  (n=60,578) | History*  (n=5,206) | Odds Ratio  (95% CI) | P-Value |
| --- | --- | --- | --- | --- | --- |
| Mildly abnormal INR | 3,231 | 2,155 | 1,076 | **7.1 (6.5-7.6)** | **<0.001** |
| Severely abnormal INR | 118 | 86 | 32 | **4.4 (2.9-6.5)** | **<0.001** |
| All abnormal INR | 3,349 | 2,241 | 1,108 | **7.0 (6.5-7.6)** | **<0.001** |
| Mildly abnormal aPTT | 4,764 | 3,839 | 925 | **3.2 (3.0-3.5)** | **<0.001** |
| Severely abnormal aPTT | 262 | 148 | 114 | **9.1 (7.1-11.7)** | **<0.001** |
| All abnormal aPTT | 5,026 | 3,987 | 1,039 | **3.5 (3.3-3.8)** | **<0.001** |
| Abnormal low platelet count | 4,115 | 2,916 | 1,199 | **5.9 (5.5-6.4)** | **<0.001** |
| Abnormal high platelet count | 1,022 | 919 | 103 | **1.3 (1.1-1.6)** | **<0.001** |

Definitions: No, number; aPTT = activated partial thromboplastin time; CI = confidence interval; INR = International Normalized Ratio; OR = operating room; RBC = red blood cell

* History = History indicative of potentially abnormal hemostasis

† Odd ratios and p values that are significant are bolded.

**Table S1F: Predictive value of “patient history indicating potentially abnormal coagulation”, abnormal hemostatic test results, both, and neither in general patients screened with all 3 hemostatic tests** (n=65,784)

| Outcome Variables | No. of patients | History* | >1 abnormal test | With history* and/or >1 abnormal test | Without history* and no abnormal coagulation tests |
| --- | --- | --- | --- | --- | --- |
| No. of patients |  | 5,206 | 10,854 | 13,711 | 52,073 |
| Perioperative RBC transfusion | 481 | 17.9% | 30.4% | 37.2% | 62.8% |
| Return to the OR | 1,584 | 13.2% | 19.5% | 27.2% | 72.8% |
| Mortality | 146 | 32.9% | 39.7% | 52.7% | 47.3% |
| Unplanned readmission | 1,243 | 15.9% | 21.4% | 28.7% | 71.3% |

Definitions: No, number

* History = History indicative of potentially abnormal hemostasis
