# Supplementary material for: Use and Utility of Hemostatic Screening in Adults Undergoing Elective, Non-Cardiac Surgery
Source: PLoS One. 2015 Dec 1;10(12):e0139139. doi: 10.1371/journal.pone.0139139 (PMC4666643; doi:10.1371/journal.pone.0139139)
Supplement: S2 Table — Table S2A. General demographics, preoperative hemostatic screening tests, patient history variables, and outcomes of interest of gynecology surgery patients (n = 33,235). Table S2B. General demographics, preoperative hemostatic screening tests, patient history variables, and outcomes of interest of gynecology surgery patients (n = 33,235). Table S2C. Outcome odds ratios by number of abnormal hemostasis test results in 6,814 gynecology surgery patients who underwent all 3 hemostasis tests. Table S2D. Outcome odds ratios by patient “history indicative of potentially abnormal hemostasis” in all gynecology surgery patients (n = 33,235). Table S2E. Abnormal screening test odds ratios by patient “history indicative of potentially abnormal hemostasis” in gynecology surgery patients screened with all 3 hemostasis tests (n = 6,814). Table S2F. Predictive value of “patient history indicating potentially abnormal coagulation”, abnormal hemostatic test results, both, and neither in gynecology patients screened with all 3 hemostatic tests (n = 6,814). (DOCX) [file pone.0139139.s002.docx]

**Table S2A: General demographics, preoperative hemostatic screening tests, patient history variables, and outcomes of interest of gynecology surgery patients** (n=33,235)

| **General demographics** | **Frequency** |
| --- | --- |
| Age, years, mean ± SD | 49 ± 11 |
| Female | 33,151 (99.8%) |
| White | 20,648 (65.3%) |
| Admitted from home | 33,12 (99.7%) |
| Partially or fully dependent functional status | 209 (0.6%) |
| ASA | |
| 1 & 2 | 26,422 (79.6%) |
| 3 & 4 | 6,791 (20.5%) |
| 5 | 1 (0.0%) |
| Prior operation within 30 days | 72 (0.4%) |
| Resident in the OR | 8,427 (41.8%) |
| **Preoperative hemostatic screening tests†** | |
| INR | 7,586 (22.8%) |
| aPTT | 7,018 (21.2%) |
| Platelet count | 30,610 (92.1%) |
| All 3 preoperative screening tests were done | 6,814 (20.5%) |
| No preoperative screening tests | 2,552 (7.7%) |
| **Patient history variables indicative of potential bleeding tendency** | |
| Bleeding disorder | 336 (1.0%) |
| Chronic steroid use | 344 (1.0%) |
| Chemotherapy | 48 (0.1%) |
| Radiation therapy | 10 (0.03%) |
| Disseminated cancer | 132 (0.4%) |
| Renal disease | 31 (0.1%) |
| Hepatic disease | 82 (0.3%) |
| History indicative of potentially abnormal hemostasis‡ | 928 (2.8%) |
| **Outcomes of interest** | |
| Perioperative RBC transfusion | 1,026 (3.1%) |
| Return to the OR | 456 (1.4%) |
| Mortality | 21 (0.06%) |
| Unplanned readmission | 677 (2.0%) |

Definitions: SD, standard deviation or standard difference; ASA = American Association of Anesthesiologists; OR, operating room; INR = International Normalized Ratio; aPTT = activated partial thromboplastin time; RBC = red blood cell;

*Procedures performed, by CPT codes, included, in descending order of frequency, are: 58150, 58260, 58571, 57288, 58552, 58550, 58570, 58541, 58180, 58262.

**Diagnoses included (ICD-9 code), in descending order of frequency, are: 218.9, 626.2, 182.0, 625.6, 218.1, 618.4, 618.5, 183.0, 617.0, 220.

† Number of patients who underwent each of the preoperative hemostatic tests within 90 days prior to surgery.

‡ Patient had one or more of the following risk factors for abnormal haemostasis: history of abnormal bleeding, self-reported family history of bleeding disorders, vitamin K deficiency, currently taking medications that pose a risk for bleeding abnormalities and/or failing to discontinue use of such medications with adequate time for normal hemostasis to be restored, chronic steroid use, chemotherapy and/or radiotherapy for cancer within 90 days prior to surgery, disseminated cancer, renal disease, and/or hepatic disease.

**Table S2B: Outcomes stratified by INR values, aPTT values, and platelet count in all gynecology surgery patients** (n=33,235)

| Test and result | No. of patients (%) | No. (%) | | | |
| --- | --- | --- | --- | --- | --- |
|  |  | Perioperative RBC transfusion | Return to the OR | Mortality | Unplanned readmission |
| **INR** | **7,586** |  |  |  |  |
| Normal | 7,382 (97.3%) | 379 (5.1%) | 115 (1.6%) | 8 (0.1%) | 193 (4.5% |
| Mildly abnormal | 196 (2.6%) | 31 (15.8%) | 9 (4.5%) | 5 (2.6%) | 9 (7.9%) |
| Severely abnormal INR | 8 (0.1%) | 0 (0.0%) | 0 (0.0%) | 0 (0.0%) | 1 (16.7%) |
| All abnormal | 204 (2.7%) | 31 (15.2%) | 9 (4.4%) | 5 (2.5%) | 10 (8.3%) |
| P-value* |  | **<0.001** | **<0.01** | **<0.001** | 0.05 |
| Sensitivity |  | 0.08 | 0.07 | 0.38 | 0.05 |
| Specificity |  | 0.98 | 0.97 | 0.97 | 0.97 |
| **aPTT** | **7,018** |  |  |  |  |
| Normal | 6,685 (95.3%) | 335 (5.3%) | 105 (1.6%) | 12 (0.2%) | 165 (4.4%) |
| Mildly abnormal | 312 (4.4%) | 21 (6.7%) | 8 (2.6%) | 1 (0.3%) | 11 (6.0%) |
| Severely abnormal | 21 (0.3%) | 4 (19.1%) | 0 (0.0%) | 0 (0.0%) | 0 (0.0%) |
| All abnormal | 333 (4.7%) | 25 (7.5%) | 8 (2.4%) | 1 (0.3%) | 11 (5.6%) |
| P-value* |  | 0.08 | 0.24 | 0.62 | 0.42 |
| Sensitivity |  | 0.07 | 0.07 | 0.08 | 0.06 |
| Specificity |  | 0.95 | 0.95 | 0.95 | 0.95 |
| **Platelet count** | **30,610** |  |  |  |  |
| Normal | 29,392 (96.0%) | 880 (3.0%) | 402 (1.4%) | 18 (0.1%) | 595 (3.4%) |
| Abnormal low | 522 (1.7%) | 25 (4.8%) | 12 (2.3%) | 1 (0.2%) | 24 (7.6%) |
| Abnormal high | 696 (2.3%) | 87 (12.5%) | 12 (1.7%) | 2 (0.3%) | 22 (6.2%) |
| P-value† |  | **0.04** | 0.07 | 0.28 | **<0.001** |
| Sensitivity‡ |  | 0.03 | 0.03 | 0.05 | 0.04 |
| Sensitivity‡ |  | 0.98 | 0.98 | 0.98 | 0.98 |

Definitions: No, number; aPTT = activated partial thromboplastin time; INR = International Normalized Ratio; RBC = red blood cell; OR = operating room

* All abnormal compared with normal. † Abnormal low platelet count compared with normal platelet count.

‡ Sensitivity and specificity are for abnormal low platelet count only. § Odd ratios and p values that are significant are bolded.

**Table S2C: Outcome odds ratios by number of abnormal hemostasis test results in 6,814 gynecology surgery patients who underwent all 3 hemostasis tests**

| Outcome Variables | No. of patients | All 3 tests are within normal range  (n=6,072) | One abnormal test  (n=649) | Odds Ratio* (95% CI) | Two or three abnormal tests  (n=93) | Odds Ratio (95% CI)* | Global P-Value† |
| --- | --- | --- | --- | --- | --- | --- | --- |
| Perioperative RBC transfusion | 366 | 281 (76.8%) | 65 (17.8%) | **2.3 (1.7-3.0)** | 20 (5.5%) | **5.6 (3.4-9.4)** | **<0.001** |
| Return to the OR | 110 | 92 (83.6%) | 16 (14.6%) | 1.6 (1.0-2.8) | 2 (1.8%) | 1.4 (0.3-5.9) | 0.17 |
| Mortality | 13 | 8 (61.5%) | 2 (15.4%) | 2.3 (0.5-11.1) | 3 (23.1%) | **25.3 (6.6-96.8)** | **<0.001** |
| Unplanned readmission | 167 | 141 (84.4%) | 22 (13.2%) | 1.6 (1.0-2.5) | 4 (2.4%) | 1.9 (0.7-5.4) | 0.09 |

CI = confidence interval; OR = operating room; RBC = red blood cell

* Odd ratios are relative to all three tests within normal range.

† Pearson's chi-square test used to compare differences in outcomes across all groups.

‡ Odd ratios and p values that are significant are bolded.

**Table S2D: Outcome odds ratios by patient “history indicative of potentially abnormal hemostasis” in all gynecology surgery patients** (n=33,235)

| Outcome Variables | No. of patients | No history*  (n=32,307) | History*  (n=928) | Odds Ratio  (95% CI) | P-Value | Sensitivity | Specificity |
| --- | --- | --- | --- | --- | --- | --- | --- |
| Perioperative RBC transfusion | 1,026 | 935 (2.9%) | 91 (9.8%) | **3.6 (2.9-4.6)** | **<0.001** | 0.09 | 0.97 |
| Return to the OR | 456 | 429 (1.3%) | 27 (2.9%) | **2.2 (1.5-3.3)** | **<0.001** | 0.06 | 0.97 |
| Mortality | 21 | 17 (0.05%) | 4 (0.4%) | **8.2 (2.8-24.5)** | **<0.001** | 0.19 | 0.97 |
| Unplanned readmission | 677 | 632 (3.3%) | 45 (7.6%) | **2.4 (1.7-3.3)** | **<0.001** | 0.07 | 0.97 |

Definitions: No, number; CI = confidence interval; RBC = red blood cell; OR = operating room

* History = History indicative of potentially abnormal hemostasis

† Odd ratios and p values that are significant are bolded.

**Table S2E: Abnormal screening test odds ratios by patient “history indicative of potentially abnormal hemostasis” in gynecology surgery patients screened with all 3 hemostasis tests** (n=6,814)

| Test Findings | No. of patients | No history*  (n=6,455) | History*  (n=359) | Odds Ratio  (95% CI) | P-Value |
| --- | --- | --- | --- | --- | --- |
| Mildly abnormal INR | 164 | 119 (72.6%) | 45 (27.4) | **7.6 (5.3-11.0)** | **<0.001** |
| Severely abnormal INR | 6 | 6 (100.0%) | 0 (0.0%) | N/C | 0.56 |
| All abnormal INR | 170 | 125 (73.5%) | 45 (26.5%) | **7.3 (5.1-10.4)** | **<0.001** |
| Mildly abnormal aPTT | 305 | 265 (86.9%) | 40 (13.1%) | **2.9 (2.1-4.2)** | **<0.001** |
| Severely abnormal aPTT | 18 | 9 (50.0%) | 9 (50.0%) | **18.4 (7.3-46.7)** | **<0.001** |
| All abnormal aPTT | 323 | 274 (84.8%) | 49 (15.25) | **3.6 (2.6-4.9)** | **<0.001** |
| Abnormal low platelet count | 158 | 128 | 30 | **4.5 (3.0-6.8)** | **<0.001** |
| Abnormal high platelet count | 191 | 169 | 22 | **2.4 (1.5-3.8)** | **<0.001** |

Definitions: No, number; aPTT = activated partial thromboplastin time; CI = confidence interval; INR = International Normalized Ratio; OR = operating room; RBC = red blood cell; N/C, not calculable.

* History = History indicative of potentially abnormal hemostasis

† Odd ratios and p values that are significant are bolded.

**Table S2F: Predictive value of “patient history indicating potentially abnormal coagulation”, abnormal hemostatic test results, both, and neither in gynecology patients screened with all 3 hemostatic tests** (n=6,814)

| Outcome Variables | No. of patients | History* | >1 abnormal test | With history* and/or >1 abnormal test | Without history* and no abnormal coagulation tests |
| --- | --- | --- | --- | --- | --- |
| No. of patients |  | 359 | 742 | 989 | 5825 |
| Perioperative RBC transfusion | 366 | 16.1% | 23.2% | 32.5% | 67.5% |
| Return to the OR | 110 | 15.5% | 16.4% | 25.5% | 74.6% |
| Mortality | 13 | 30.8% | 38.5% | 46.2% | 53.9% |
| Unplanned readmission | 167 | 14.4% | 15.6% | 27.0% | 73.1% |

Definitions: No, number

* History = History indicative of potentially abnormal hemostasis
