# Supplementary material for: Use and Utility of Hemostatic Screening in Adults Undergoing Elective, Non-Cardiac Surgery
Source: PLoS One. 2015 Dec 1;10(12):e0139139. doi: 10.1371/journal.pone.0139139 (PMC4666643; doi:10.1371/journal.pone.0139139)
Supplement: S3 Table — Table S3A. General demographics, preoperative hemostatic screening tests, patient history variables, and outcomes of interest of neurosurgery patients (n = 24,453). Table S3B. Outcomes stratified by INR values, aPTT values, and platelet count in all neurosurgery patients (n = 24,453). Table S3C. Outcome odds ratios by number of abnormal hemostasis test results in 14,500 neurosurgery patients who underwent all 3 hemostasis tests. Table S3D. Outcome odds ratios by patient “history indicative of potentially abnormal hemostasis” in all neurosurgery patients (n = 24,453). Table S3E. Abnormal screening test odds ratios by patient “history indicative of potentially abnormal hemostasis” in neurosurgery patients screened with all 3 hemostasis tests (n = 14,500). Table S3F. Predictive value of “patient history indicating potentially abnormal coagulation”, abnormal hemostatic test results, both, and neither in neurosurgery patients screened with all 3 hemostatic tests (n = 14,500). (DOCX) [file pone.0139139.s003.docx]

**Table S3A: General demographics, preoperative hemostatic screening tests, patient history variables, and outcomes of interest of neurosurgery patients** (n=24,453)

| **General demographics** | **Frequency** |
| --- | --- |
| Age, years, mean ± SD | 56 ± 14 |
| Female | 12,119 (49.6%) |
| White | 19,346 (82.2%) |
| Admitted from home | 23,972 (98.0%) |
| Partially or fully dependent functional status | 765 (3.1%) |
| ASA | |
| 1 & 2 | 14,221 (58.2%) |
| 3 & 4 | 10,199 (41.8%) |
| 5 | 2 (0.01%) |
| Prior operation within 30 days | 80 (0.6%) |
| Resident in OR | 4,601 (33.0%) |
| **Preoperative hemostatic screening tests†** | |
| INR | 16,315 (66.7%) |
| aPTT | 14,790 (60.5%) |
| Platelet count | 22,123 (90.5%) |
| All 3 preoperative screening tests were done | 14,500 (59.3%) |
| No preoperative screening tests | 2,114 (8.7%) |
| **Patient history variables indicative of potential bleeding tendency** | |
| Bleeding disorder | 368 (1.5%) |
| Chronic steroid use | 1,236 (5.1%) |
| Chemotherapy | 85 (0.4%) |
| Radiation therapy | 41 (0.2%) |
| Disseminated cancer | 893 (3.7%) |
| Renal disease | 43 (0.2%) |
| Hepatic disease | 3 (0.01%) |
| History indicative of potentially abnormal hemostasis‡ | 2,179 (8.9%) |
| **Outcomes of interest** | |
| Perioperative RBC transfusion | 1,071 (4.4%) |
| Return to the OR | 588 (2.4%) |
| Mortality | 77 (0.3%) |
| Unplanned readmission | 726 (3.0%) |

Definitions: SD, standard deviation or standard difference; ASA = American Association of Anesthesiologists; OR, operating room; INR = International Normalized Ratio; aPTT = activated partial thromboplastin time; RBC = red blood cell;

*Procedures performed, by CPT codes, included, in descending order of frequency, are: 63030, 63047, 61510, 22551, 22612, 63075, 22554, 22630, 61512, 63042.

**Diagnoses included (ICD-9 code), in descending order of frequency, are: 722.10, 724.01, 722.0, 721.1, 722.52, 721.3, 225.2, 721.0, 198.3, 738.4. 278.0, 550.90, 574.10, 553.21, 174.9, 553.1, 562.11, 233.0, 574.20, 540.9.

† Number of patients who underwent each of the preoperative hemostatic tests within 90 days prior to surgery.

‡ Patient had one or more of the following risk factors for abnormal haemostasis: history of abnormal bleeding, self-reported family history of bleeding disorders, vitamin K deficiency, currently taking medications that pose a risk for bleeding abnormalities and/or failing to discontinue use of such medications with adequate time for normal hemostasis to be restored, chronic steroid use, chemotherapy and/or radiotherapy for cancer within 90 days prior to surgery, disseminated cancer, renal disease, and/or hepatic disease.

**Table S3B: Outcomes stratified by INR values, aPTT values, and platelet count in all neurosurgery patients** (n=24,453)

| Test and result | No. of patients (%) | No. (%) | | | |
| --- | --- | --- | --- | --- | --- |
|  |  | Perioperative RBC transfusion | Return to the OR | Mortality | Unplanned readmission |
| **INR** | **16,315** |  |  |  |  |
| Normal | 16,000 (98.1%) | 744 (4.7%) | 385 (2.4%) | 57 (0.4%) | 529 (5.0%) |
| Mildly abnormal | 289 (1.8%) | 22 (7.6%) | 10 (3.5%) | 11 (3.8%) | 11 (6.2%) |
| Severely abnormal INR | 26 (0.2%) | 5 (19.2%) | 4 (15.4%) | 0 (0.0%) | 2 (13.3%) |
| All abnormal | 315 (1.9%) | 27 (8.6%) | 14 (4.4%) | 11 (3.5%) | 13 (6.7%) |
| P-value* |  | **0.001** | **0.02** | **<0.001** | 0.26 |
| Sensitivity |  | 0.04 | 0.04 | 0.16 | 0.02 |
| Specificity |  | 0.98 | 0.98 | 0.98 | 0.98 |
| **aPTT** | **14,790** |  |  |  |  |
| Normal | 14,179 (95.9%) | 686 (4.6%) | 332 (2.3%) | 51 (0.4%) | 453 (4.8%) |
| Mildly abnormal | 593 (4.0%) | 34 (5.7%) | 23 (3.9%) | 6 (1.0%) | 23 (5.8%) |
| Severely abnormal | 18 (0.1%) | 1 (5.6%) | 1 (5.6%) | 0 (0.0%) | 2 (16.7%) |
| All abnormal | 611 (4.1%) | 35 (5.7%) | 24 (3.9%) | 6 (1.0%) | 25 (6.1%) |
| P-value* |  | 0.32 | **0.01** | **0.02** | 0.24 |
| Sensitivity |  | 0.05 | 0.07 | 0.11 | 0.05 |
| Specificity |  | 0.96 | 0.96 | 0.96 | 0.96 |
| **Platelet count** | **22,123** |  |  |  |  |
| Normal | 20,905 (94.5%) | 884 (4.2%) | 500 (2.4%) | 60 (0.3%) | 614 (4.6%) |
| Abnormal low | 1,011 (4.6%) | 75 (7.4%) | 34 (3.4%) | 15 (1.5%) | 48 (7.2%) |
| Abnormal high | 207 (0.9%) | 14 (6.8%) | 5 (2.4%) | 1 (0.5%) | 4 (3.0%) |
| P-value† |  | **<0.001** | 0.05 | **<0.001** | **<0.01** |
| Sensitivity‡ |  | 0.08 | 0.06 | 0.20 | 0.07 |
| Sensitivity‡ |  | 0.96 | 0.95 | 0.95 | 0.95 |

Definitions: No, number; = activated partial thromboplastin time; INR = International Normalized Ratio; RBC = red blood cell; OR = operating room

* All abnormal compared with normal. † Abnormal low platelet count compared with normal platelet count.

‡ Sensitivity and specificity are for abnormal low platelet count only. § Odd ratios and p values that are significant are bolded.

**Table S3C: Outcome odds ratios by number of abnormal hemostasis test results in 14,500 neurosurgery patients who underwent all 3 hemostasis tests**

| Outcome Variables | No. of patients | All 3 tests are within normal range  (n=12,994) | One abnormal test  (n=1,363) | Odds Ratio* (95% CI) | Two or three abnormal tests  (n=143) | Odds Ratio (95% CI)* | Global P-Value† |
| --- | --- | --- | --- | --- | --- | --- | --- |
| Perioperative RBC transfusion | 706 | 602 (4.6%) | 86 (6.3%) | **1.4 (1.1-1.8)** | 18 (12.6%) | **3.0 (1.8-4.9)** | **<0.001** |
| Return to the OR | 347 | 296 (2.3%) | 44 (3.2%) | 1.4 (1.0-2.0**)** | 7 (4.9%) | 2.2 (1.0-4.8) | **0.01** |
| Mortality | 57 | 38 (0.3%) | 14 (1.0%) | **3.5 (1.9-6.5)** | 5 (3.5%) | **12.4 (4.8-31.9)** | **<0.001** |
| Unplanned readmission | 471 | 405 (4.7%) | 60 (6.5%) | **1.4 (1.1-1.8)** | 6 (7.1%) | 1.5 (0.7-3.5) | **0.04** |

Definitions: No, number; CI = confidence interval; OR = operating room; RBC = red blood cell

* Odd ratios are relative to all three tests within normal range.

† Pearson's chi-square test used to compare differences in outcomes across all groups.

‡ Odd ratios and p values that are significant are bolded.

**Table S3D: Outcome odds ratios by patient “history indicative of potentially abnormal hemostasis” in all neurosurgery patients** (n=24,453)

| Outcome Variables | No. of patients | No history*  (n=22,274) | History*  (n=2,179) | Odds Ratio  (95% CI) | P-Value | Sensitivity | Specificity |
| --- | --- | --- | --- | --- | --- | --- | --- |
| Perioperative RBC transfusion | 1,071 | 930 (4.2%) | 141 (6.5%) | **1.6 (1.3-1.9)** | **<0.001** | 0.13 | 0.91 |
| Return to the OR | 588 | 499 (2.2%) | 89 (4.1%) | **1.9 (1.4-2.3)** | **<0.001** | 0.15 | 0.91 |
| Mortality | 77 | 33 (0.2%) | 44 (2.0%) | **13.9 (8.8-21.9)** | **<0.001** | 0.57 | 0.91 |
| Unplanned readmission | 726 | 577 (4.1%) | 149 (9.7%) | **2.5 (2.1-.3.1)** | **<0.001** | 0.21 | 0.91 |

Definitions: No, number; CI = confidence interval; RBC = red blood cell; OR = operating room

* History = History indicative of potentially abnormal hemostasis

† Odd ratios and p values that are significant are bolded.

**Table S3E: Abnormal screening test odds ratios by patient “history indicative of potentially abnormal hemostasis” in neurosurgery patients screened with all 3 hemostasis tests** (n=14,500)

| Test Findings | No. of patients | No history*  (n=13,046) | History*  (n=1,454) | Odds Ratio  (95% CI) | P-Value |
| --- | --- | --- | --- | --- | --- |
| Mildly abnormal INR | 240 | 165 | 75 | **4.2 (3.2-5.6)** | **<0.001** |
| Severely abnormal INR | 22 | 17 | 5 | 2.6 (1.0-7.2) | 0.05 |
| All abnormal INR | 262 | 182 | 80 | **4.1 (3.1-5.4)** | **<0.001** |
| Mildly abnormal aPTT | 575 | 492 | 83 | **1.5 (1.2-2.0)** | **<0.001** |
| Severely abnormal aPTT | 17 | 13 | 4 | 2.8 (0.9-8.5) | 0.06 |
| All abnormal aPTT | 592 | 505 | 87 | **1.6 (1.3-2.0)** | **<0.001** |
| Abnormal low platelet count | 670 | 521 | 149 | **2.7 (2.3-3.3)** | **<0.001** |
| Abnormal high platelet count | 143 | 110 | 33 | **2.6 (1.8-4.0)** | **<0.001** |

Definitions: No, number; aPTT = activated partial thromboplastin time; CI = confidence interval; INR = International Normalized Ratio; OR = operating room; RBC = red blood cell

* History = History indicative of potentially abnormal hemostasis

† Odd ratios and p values that are significant are bolded.

**Table S3F: Predictive value of “patient history indicating potentially abnormal coagulation”, abnormal hemostatic test results, both, and neither in neurosurgery patients screened with all 3 hemostatic tests** (n= 14,500)

| Outcome Variables | No. of patients | History* | >1 abnormal test | With history* and/or >1 abnormal test | Without history* and no abnormal coagulation tests |
| --- | --- | --- | --- | --- | --- |
| No. of patients |  | 1,454 | 1,506 | 2,653 | 11,847 |
| Perioperative RBC transfusion | 706 | 13.5% | 14.7% | 23.9% | 76.1% |
| Return to the OR | 347 | 15.9% | 14.7% | 25.7% | 74.4% |
| Mortality | 57 | 66.7% | 33.3% | 75.4% | 24.6% |
| Unplanned readmission | 471 | 22.5% | 14.0% | 32.7% | 67.3% |

Definitions: No, number

* History = History indicative of potentially abnormal hemostasis
