# Supplementary material for: Use and Utility of Hemostatic Screening in Adults Undergoing Elective, Non-Cardiac Surgery
Source: PLoS One. 2015 Dec 1;10(12):e0139139. doi: 10.1371/journal.pone.0139139 (PMC4666643; doi:10.1371/journal.pone.0139139)
Supplement: S4 Table — Table S4A. General demographics, preoperative hemostatic screening tests, patient history variables, and outcomes of interest of orthopedic surgery patients (n = 90,627). Table S4B. Outcomes stratified by INR values, aPTT values, and platelet count in all orthopedic surgery patients (n = 90,627). Table S4C. Outcome odds ratios by number of abnormal hemostasis test results in 41,445 orthopedic surgery patients who underwent all 3 hemostasis tests.Table S4D. Outcome odds ratios by patient “history indicative of potentially abnormal hemostasis” in all orthopedic surgery patients (n = 90,627). Table S4E. Abnormal screening test odds ratios by patient “history indicative of potentially abnormal hemostasis” in orthopedic surgery patients screened with all 3 hemostasis tests (n = 41,445). Table S4F. Predictive value of “patient history indicating potentially abnormal coagulation”, abnormal hemostatic test results, both, and neither in orthopedic surgery patients screened with all 3 hemostatic tests (n = 41,445). (DOCX) [file pone.0139139.s004.docx]

**Table S4A: General demographics, preoperative hemostatic screening tests, patient history variables, and outcomes of interest of orthopedic surgery patients** (n=90,627)

| **General demographics** | **Frequency** |
| --- | --- |
| Age, years, mean ± SD | 65 ± 12 |
| Female | 53,249 (58.8%) |
| White | 69,594 (81.6%) |
| Admitted from home | 89,584 (98.9%) |
| Partially or fully dependent functional status | 3,223 (3.6%) |
| ASA | |
| 1 & 2 | 51,559 (57.0%) |
| 3 & 4 | 38,944 (43.0%) |
| 5 | 5 (0.01%) |
| Prior operation within 30 days | 110 (0.2%) |
| Resident in OR | 12,459 (26.6%) |
| **Preoperative hemostatic screening tests†** | |
| INR | 57,242 (63.2%) |
| aPTT | 42,309 (46.7%) |
| Platelet count | 82,163 (90.7%) |
| All 3 preoperative screening tests were done | 41,445 (45.7%) |
| No preoperative screening tests | 7,248 (8.0%) |
| **Patient history variables indicative of potential bleeding tendency** | |
| Bleeding disorder | 2,541 (2.8%) |
| Chronic steroid use | 2,197 (2.4%) |
| Chemotherapy | 76 (0.1%) |
| Radiation therapy | 19 (0.02%) |
| Disseminated cancer | 151 (0.2%) |
| Renal disease | 167 (0.2%) |
| Hepatic disease | 32 (0.04%) |
| History indicative of potentially abnormal hemostasis‡ | 4,960 (5.5%) |
| **Outcomes of interest** | |
| Perioperative RBC transfusion | 11,968 (13.2%) |
| Return to the OR | 1,233 (1.4%) |
| Mortality | 274 (0.3%) |
| Unplanned readmission | 2,295 (2.5%) |

Definitions: SD, standard deviation or standard difference; ASA = American Association of Anesthesiologists; OR, operating room; INR = International Normalized Ratio; aPTT = activated partial thromboplastin time; RBC = red blood cell;

*Procedures performed, by CPT codes, included, in descending order of frequency, are: 27447, 27130, 29881, 2927, 29880, 29888, 29826, 63030, 27236, 27245.

**Diagnoses included (ICD-9 code), in descending order of frequency, are: 715.36, 715.35, 715.96, 836.0, 715.95, 820.21, 715.15, 722.10, 840.4.

† Number of patients who underwent each of the preoperative hemostatic tests within 90 days prior to surgery.

‡ Patient had one or more of the following risk factors for abnormal haemostasis: history of abnormal bleeding, self-reported family history of bleeding disorders, vitamin K deficiency, currently taking medications that pose a risk for bleeding abnormalities and/or failing to discontinue use of such medications with adequate time for normal hemostasis to be restored, chronic steroid use, chemotherapy and/or radiotherapy for cancer within 90 days prior to surgery, disseminated cancer, renal disease, and/or hepatic disease.

**Table S4B: Outcomes stratified by INR values, aPTT values, and platelet count in all orthopedic surgery patients** (n=90,627)

| Test and result | No. of patients (%) | No. (%) | | | |
| --- | --- | --- | --- | --- | --- |
|  |  | Perioperative RBC transfusion | Return to the OR | Mortality | Unplanned readmission |
| **INR** | **57,242** |  |  |  |  |
| Normal | 55,105 (96.3%) | 8,442 (15.3%) | 716 (1.3%) | 185 (0.3%) | 1,500 (3.9%) |
| Mildly abnormal | 2,014 (3.5%) | 417 (20.7%) | 47 (2.3%) | 35 (1.7%) | 124 (9.4%) |
| Severely abnormal INR | 123 (0.2%) | 26 (21.14%) | 8 (6.5%) | 2 (2.4%) | 11 (14.7%) |
| All abnormal | 2137 (3.7%) | 443 (20.7%) | 55 (2.6%) | 38 (1.8%) | 135 (9.7%) |
| P-value* |  | **<0.001** | **<0.001** | **<0.001** | **<0.001** |
| Sensitivity |  | 0.05 | 0.07 | 0.17 | 0.08 |
| Specificity |  | 0.97 | 0.96 | 0.96 | 0.97 |
| **aPTT** | **42,309** |  |  |  |  |
| Normal | 39,928 (94.4%) | 5,907 (14.8%) | 545 (1.4%) | 149 (0.4%) | 1,054 (3.9%) |
| Mildly abnormal | 2,324 (5.5%) | 443 (19.1%) | 33 (1.4%) | 17 (0.7%) | 113 (6.9%) |
| Severely abnormal | 57 (0.1%) | 11 (19.3%) | 1 (1.8%) | 0 (0.0%) | 2 (5.1%) |
| All abnormal | 2,381 (5.6%) | 454 (19.1%) | 34 (1,4%) | 17 (0.7%) | 115 (6.8%) |
| P-value* |  | **<0.001** | 0.80 | **<0.01** | **<0.001** |
| Sensitivity |  | 0.07 | 0.06 | 0.10 | 0.10 |
| Specificity |  | 0.95 | 0.94 | 0.94 | 0.94 |
| **Platelet count** | **82,163** |  |  |  |  |
| Normal | 77,321 (94.1%) | 10,431 (13.5%) | 1,041 (1.4%) | 210 (0.3%) | 1,979 (3.8%) |
| Abnormal low | 4,117 (5.0%) | 833 (20.2%) | 74 (1.8%) | 54 (1.3%) | 151 (5.3%) |
| Abnormal high | 725 (0.9%) | 136 (18.8%) | 16 (2.2%) | 2 (0.3%) | 31 (6.8%) |
| P-value† |  | **<0.001** | **0.02** | **<0.001** | **<0.001** |
| Sensitivity‡ |  | 0.07 | 0.07 | 0.20 | 0.07 |
| Sensitivity‡ |  | 0.95 | 0.95 | 0.95 | 0.95 |

Definitions: No, number; aPTT = activated partial thromboplastin time; INR = International Normalized Ratio; RBC = red blood cell; OR = operating room

* All abnormal compared with normal.

† Abnormal low platelet count compared with normal platelet count.

‡ Sensitivity and specificity are for abnormal low platelet count only.

§ Odd ratios and p values that are significant are bolded.

**Table S4C: Outcome odds ratios by number of abnormal hemostasis test results in 41,445 orthopedic surgery patients who underwent all 3 hemostasis tests**

| Outcome Variables | No. of patients | All 3 tests are within normal range  (n=36,054) | One abnormal test  (n=4,541) | Odds Ratio* (95% CI) | Two or three abnormal tests  (n=850) | Odds Ratio (95% CI)* | Global P-Value† |
| --- | --- | --- | --- | --- | --- | --- | --- |
| Perioperative RBC transfusion | 6,267 | 5,180 (82.7%) | 878 (14.0%) | **1.4 (1.3-1.5)** | 209 (3.3%) | **1.9 (1.7-2.3)** | **<0.001** |
| Return to the OR | 561 | 470 (83.8%) | 71 (12.7%) | 1.2 (0.9-1.5) | 30 (3.6%) | **1.8 (1.2-2.9)** | **0.01** |
| Mortality | 166 | 108 (65.1%) | 42 (25.3%) | **3.1 (2.2-4.4)** | 16 (9.6%) | **6.4 (3.8-10.8)** | **<0.001** |
| Unplanned readmission | 1,140 | 901 (79.0%) | 181 (15.9%) | **1.6 (1.4-1.9)** | 58 (5.1%) | **2.9 (2.2-3.8)** | **<0.001** |

Definitions: No, number; CI = confidence interval; OR = operating room; RBC = red blood cell

* Odd ratios are relative to all three tests within normal range.

† Pearson's chi-square test used to compare differences in outcomes across all groups.

‡ Odd ratios and p values that are significant are bolded.

**Table S4D: Outcome odds ratios by patient “history indicative of potentially abnormal hemostasis” in all orthopedic surgery patients** (n=90,627)

| Outcome Variables | No. of patients | No history*  (n=85,667) | History*  (n=4,960) | Odds Ratio  (95% CI) | P-Value | Sensitivity | Specificity |
| --- | --- | --- | --- | --- | --- | --- | --- |
| Perioperative RBC transfusion | 11,968 | 10,990 (12.8%) | 978 (19.7%) | **1.7 (1.6-1.8)** | **<0.001** | 0.08 | 0.95 |
| Return to the OR | 1,233 | 1,119 (1.3%) | 114 (2.3%) | **1.8 (1.5-2.2)** | **<0.001** | 0.09 | 0.95 |
| Mortality | 274 | 201 (0.2%) | 73 (1.5%) | **6.4 (4.8-8.3)** | **<0.001** | 0.27 | 0.95 |
| Unplanned readmission | 2,295 | 2,026 (3.5%) | 269 (7.9%) | **2.3 (2.1-2.7)** | **<0.001** | 0.12 | 0.96 |

Definitions: NO, number; CI = confidence interval; RBC = red blood cell; OR = operating room

* History = History indicative of potentially abnormal hemostasis

† Odd ratios and p values that are significant are bolded.

**Table S4E: Abnormal screening test odds ratios by patient “history indicative of potentially abnormal hemostasis” in orthopedic surgery patients screened with all 3 hemostasis tests** (n=41,445)

| Test Findings | No. of patients | No history*  (n=2,635) | History*  (n=38,810) | Odds Ratio  (95% CI) | P-Value |
| --- | --- | --- | --- | --- | --- |
| Mildly abnormal INR | 1,312 | 869 | 443 | **8.8 (7.8-10.0)** | **<0.001** |
| Severely abnormal INR | 84 | 64 | 20 | **4.6 (2.8-7.7)** | **<0.001** |
| All abnormal INR | 1,396 | 933 | 463 | **8.7 (7.7-9.7)** | **<0.001** |
| Mildly abnormal aPTT | 2,278 | 1,875 | 403 | **3.6 (3.2-4.0)** | **<0.001** |
| Severely abnormal aPTT | 54 | 34 | 20 | **8.7 (5.0-15.2)** | **<0.001** |
| All abnormal aPTT | 2,332 | 1,909 | 423 | **3.7 (3.3-4.1)** | **<0.001** |
| Abnormal low platelet count | 2,248 | 1,859 | 389 | **3.4 (3.1-3.9)** | **<0.001** |
| Abnormal high platelet count | 377 | 332 | 45 | **2.0 (1.5-2.8)** | **<0.001** |

Definitions: No, number; aPTT = activated partial thromboplastin time; CI = confidence interval; INR = International Normalized Ratio; OR = operating room; RBC = red blood cell

* History = History indicative of potentially abnormal hemostasis

† Odd ratios and p values that are significant are bolded.

**Table S4F: Predictive value of “patient history indicating potentially abnormal coagulation”, abnormal hemostatic test results, both, and neither in orthopedic surgery patients screened with all 3 hemostatic tests** (n=41,445)

| Outcome Variables | No. of patients | History* | >1 abnormal test | With history* and/or >1 abnormal test | Without history* and no abnormal coagulation tests |
| --- | --- | --- | --- | --- | --- |
| No. of patients |  | 2,635 | 5,391 | 7,065 | 34,380 |
| Perioperative RBC transfusion | 6,267 | 9.1% | 17.3% | 22.4% | 77.6% |
| Return to the OR | 561 | 10.9% | 16.2% | 22.8% | 77.2% |
| Mortality | 166 | 30.7% | 34.9% | 49.4% | 50.6% |
| Unplanned readmission | 1,140 | 14.0% | 21.0% | 28.5% | 82.9% |

* History = History indicative of potentially abnormal hemostasis
