# Supplementary material for: Use and Utility of Hemostatic Screening in Adults Undergoing Elective, Non-Cardiac Surgery
Source: PLoS One. 2015 Dec 1;10(12):e0139139. doi: 10.1371/journal.pone.0139139 (PMC4666643; doi:10.1371/journal.pone.0139139)
Supplement: S5 Table — Table S5A. General demographics, preoperative hemostatic screening tests, patient history variables, and outcomes of interest of otolaryngology patients (n = 14,706). Table S5B. Outcomes stratified by INR values, aPTT values, and platelet count in all otolaryngology surgery patients (n = 14,706). Table S5C. Outcome odds ratios by number of abnormal hemostasis test results in 4,006 otolaryngology surgery patients who underwent all 3 hemostasis tests. Table S5D. Outcome odds ratios by patient “history indicative of potentially abnormal hemostasis” in all otolaryngology patients (n = 14,706). Table S5E. Abnormal screening test odds ratios by patient “history indicative of potentially abnormal hemostasis” in otolaryngology patients screened with all 3 hemostasis tests (n = 4,006). Table S5F. Predictive value of “patient history indicating potentially abnormal coagulation”, abnormal hemostatic test results, both, and neither in otolaryngology patients screened with all 3 hemostatic tests (n = 4,006). (DOCX) [file pone.0139139.s005.docx]

**Table S5A: General demographics, preoperative hemostatic screening tests, patient history variables, and outcomes of interest of otolaryngology patients** (n=14,706)

| **General demographics** | **Frequency** |
| --- | --- |
| Age, years, mean ± SD | 43 ± 17 |
| Female | 9,858 (67.0%) |
| White | 9,424 (69.3%) |
| Admitted from home | 14,677 (99.8%) |
| Partially or fully dependent functional status | 61 (0.4%) |
| ASA | |
| 1 & 2 | 11,502 (78.4%) |
| 3 & 4 | 3,176 (21.6%) |
| 5 | - |
| Prior operation within 30 days | 48 (0.5%) |
| Resident in OR | 2,896 (30.7%) |
| **Preoperative hemostatic screening tests†** | |
| INR | 4,493 (30.6%) |
| aPTT | 4,174 (28.4%) |
| Platelet count | 9,302 (63.3%) |
| All 3 preoperative screening tests were done | 4,006 (27.2%) |
| No preoperative screening tests | 5,258 (35.8%) |
| **Patient history variables indicative of potential bleeding tendency** | |
| Bleeding disorder | 103 (0,7%) |
| Chronic steroid use | 181 (1.2%) |
| Chemotherapy | 19 (0.1%) |
| Radiation therapy | 9 (0.06%) |
| Disseminated cancer | 92 (0.6%) |
| Renal disease | 16 (0.1%) |
| Hepatic disease | 2 (0.01%) |
| History indicative of potentially abnormal hemostasis‡ | 398 (2.7%) |
| **Outcomes of interest** | |
| Perioperative RBC transfusion | 9 (0.06%) |
| Return to the OR | 362 (2.5%) |
| Mortality | 5 (0.03%) |
| Unplanned readmission | 265 (1.8%) |

Definitions: SD, standard deviation or standard difference; ASA = American Association of Anesthesiologists; OR, operating room; INR = International Normalized Ratio; aPTT = activated partial thromboplastin time; RBC = red blood cell;

*Procedures performed, by CPT codes, included, in descending order of frequency, are: 42826, 60240, 60220, 42821, 42415, 38724, 42145, 60500, 69631, 60252.

**Diagnoses included (ICD-9 code), in descending order of frequency, are: 474.00, 193, 210.2, 241.1, 327.23, 226, 241.0, 474.11, 384.20, 784.2.

† Number of patients who underwent each of the preoperative hemostatic tests within 90 days prior to surgery.

‡ Patient had one or more of the following risk factors for abnormal haemostasis: history of abnormal bleeding, self-reported family history of bleeding disorders, vitamin K deficiency, currently taking medications that pose a risk for bleeding abnormalities and/or failing to discontinue use of such medications with adequate time for normal hemostasis to be restored, chronic steroid use, chemotherapy and/or radiotherapy for cancer within 90 days prior to surgery, disseminated cancer, renal disease, and/or hepatic disease.

| Test and result | No. of patients (%) | No. (%) | | | |
| --- | --- | --- | --- | --- | --- |
|  |  | Perioperative RBC transfusion | Return to the OR | Mortality | Unplanned readmission |
| **INR** | **4,493** |  |  |  |  |
| Normal | 4403 (98.0%) | 2 (0.05%) | 101 (2.3%) | 2 (0.05%) | 61 (2.5%) |
| Mildly abnormal | 86 (1.9%) | 0 (0.0%) | 4 (4.7%) | 0 (0.0%) | 4 (9.3%) |
| Severely abnormal INR | 4 (0.1%0 | 0 (0.0%) | 0 (0.0%) | 0 (0.0%) | 0 (0.0%) |
| All abnormal | 90 (2.0%) | 0 (0.0%) | 4 (4.4%) | 0 (0.0%) | 4 (8.7%) |
| P-value* |  | 0.98 | 0.34 | 0.98 | **0.02** |
| Sensitivity |  | 0.00 | 0.04 | 0.00 | 0.06 |
| Specificity |  | 0.98 | 0.98 | 0.98 | 0.98 |
| **aPTT** | **4,147** |  |  |  |  |
| Normal | 3,973 (95.2%) | 2 (0.05%) | 91 (2.3%) | 2 (0.05%) | 47 (2.2%) |
| Mildly abnormal | 195 (5.7%) | 0 (0.0%) | 8 (4.1%) | 0 (0.0%) | 11 (9.5%) |
| Severely abnormal | 6 (0.1%) | 0 (0.0%) | 0 (0.0%) | 0 (0.0%) | 0 (0.0%) |
| All abnormal | 201 (4.8%) | 0 (0.0%) | 8 (4.0%) | 0 (0.0%) | 11 (9.4%) |
| P-value* |  | 0.95 | 0.25 | 0.95 | **<0.001** |
| Sensitivity |  | 0.00 | 0.08 | 0.00 | 0.19 |
| Specificity |  | 0.95 | 0.95 | 0.95 | 0.95 |
| **Platelet count** | **9,302** |  |  |  |  |
| Normal | 8,936 (96.1%) | 5 (0.06%) | 211 (2.4%) | 4 (0.04%) | 154 (3.0%) |
| Abnormal low | 277 (3.0%) | 0 (0.0%) | 7 (2.5%) | 0 (0.0%) | 11 (6.8%) |
| Abnormal high | 89 (1.0%) | 0 (0.0%) | 2 (2.3%) | 1 (1.1%) | 5 (9.6%) |
| P-value† |  | 0.90 | 0.98 | **<0.001** | **<0.001** |
| Sensitivity‡ |  | 0.00 | 0.03 | 0.00 | 0.06 |
| Sensitivity‡ |  | 0.97 | 0.97 | 0.97 | 0.97 |

**Table S5B: Outcomes stratified by INR values, aPTT values, and platelet count in all otolaryngology surgery patients** (n=14,706)

Definitions: NO, number; aPTT = activated partial thromboplastin time; INR = International Normalized Ratio; RBC = red blood cell; OR = operating room

* All abnormal compared with normal. † Abnormal low platelet count compared with normal platelet count.

‡ Sensitivity and specificity are for abnormal low platelet count only. § Odd ratios and p values that are significant are bolded.

**Table S5C: Outcome odds ratios by number of abnormal hemostasis test results in 4,006 otolaryngology surgery patients who underwent all 3 hemostasis tests**

| Outcome Variables | No. of patients | All 3 tests are within normal range  (n=3,620) | One abnormal test  (n=354) | Odds Ratio* (95% CI) | Two or three abnormal tests  (n=32) | Odds Ratio (95% CI)* | Global P-Value† |
| --- | --- | --- | --- | --- | --- | --- | --- |
| Perioperative RBC transfusion | 2 | 2 (0.06%) | 0 (0.0%) | **NA** | 0 (0.0%) | **NA** | 0.90 |
| Return to the OR | 91 | 76 (2.1%) | 14 (4.0%) | **1.9 (1.1-3.4)** | 1 (3.1%) | 1.5 (0.2-11.2) | 0.08 |
| Mortality | 2 | 3 (0.06%) | 0 (0.0%) | N/C | 0 (0.0%) | **N/C** | 0.90 |
| Unplanned readmission | 54 | 40 (2.1%) | 10 (5.2%) | **2.6 (1.3-5.3)** | 4 (17.4%) | **10.0 (3.3-30.8)** | **<0.001** |

Definitions: No, number; CI = confidence interval; OR = operating room; RBC = red blood cell; not calculable

* Odd ratios are relative to all three tests within normal range.

† Pearson's chi-square test used to compare differences in outcomes across all groups.

‡ Odd ratios and p values that are significant are bolded.

**Table S5D: Outcome odds ratios by patient “history indicative of potentially abnormal hemostasis” in all otolaryngology patients** (n=14,706)

| Outcome Variables | No. of patients | No history*  (n=14,308) | History*  (n=398) | Odds Ratio  (95% CI) | P-Value | Sensitivity | Specificity |
| --- | --- | --- | --- | --- | --- | --- | --- |
| Perioperative RBC transfusion | 9 | 7 (0.05%) | 2 (0.5%) | **10.3 (2.1-49.8)** | **<0.001** | 0.22 | 0.97 |
| Return to the OR | 362 | 343 (2.4%) | 19 (4.8%) | **2.0 (1.3-3.3)** | **<0.01** | 0.05 | 0.97 |
| Mortality | 5 | 4 (0.03%) | 1 (0.3%) | **9.0 (1.0-80.8)** | **0.02** | 0.20 | 0.97 |
| Unplanned readmission | 265 | 252 (3.0%) | 13 (5.3%) | **1.8 (1.0-3.2)** | **0.04** | 0.05 | 0.97 |

Definitions: No, number; CI = confidence interval; RBC = red blood cell; OR = operating room

* History = History indicative of potentially abnormal hemostasis

† Odd ratios and p values that are significant are bolded.

**Table S5E: Abnormal screening test odds ratios by patient “history indicative of potentially abnormal hemostasis” in otolaryngology patients screened with all 3 hemostasis tests** (n=4,006)

| Test Findings | No. of patients | No history*  (n=3,871) | History*  (n=135) | Odds Ratio  (95% CI) | P-Value |
| --- | --- | --- | --- | --- | --- |
| Mildly abnormal INR | 58 | 49 | 9 | **5.6 (2.7-11.6)** | **<0.001** |
| Severely abnormal INR | 2 | 2 | 0 | N/C | 0.79 |
| All abnormal INR | 60 | 51 | 9 | **5.4 (2.6-11.1)** | **<0.001** |
| Mildly abnormal aPTT | 191 | 181 | 10 | 1.6 (0.8-3.2) | 0.14 |
| Severely abnormal aPTT | 6 | 4 | 2 | **14.5 (2.6-80.1)** | **<0.001** |
| All abnormal aPTT | 197 | 185 | 12 | **1.9 (1.1-3.6)** | **0.03** |
| Abnormal low platelet count | 122 | 110 | 12 | **3.3 (1.8-6.2)** | **<0.001** |
| Abnormal high platelet count | 44 | 42 | 2 | 1.4 (0.3-5.7) | 0.66 |

Definitions: No, number; aPTT = activated partial thromboplastin time; CI = confidence interval; INR = International Normalized Ratio; OR = operating room; RBC = red blood cell; N/C, not calculable

* History = History indicative of potentially abnormal hemostasis

† Odd ratios and p values that are significant are bolded.

**Table S5F: Predictive value of “patient history indicating potentially abnormal coagulation”, abnormal hemostatic test results, both, and neither in otolaryngology patients screened with all 3 hemostatic tests** (n=4,006)

| Outcome Variables | No. of patients | History* | >1 abnormal test | With history* and/or >1 abnormal test | Without history* and no abnormal coagulation tests |
| --- | --- | --- | --- | --- | --- |
| No. of patients |  | 135 | 386 | 488 | 3518 |
| Perioperative RBC transfusion | 2 | 0.0% | 0.0% | 0.0% | 100.0% |
| Return to the OR | 91 | 7.7% | 16.5% | 22.0% | 78.0% |
| Mortality | 2 | 0.0% | 0.0% | 0.0% | 100.0% |
| Unplanned readmission | 54 | 3.7% | 25.9% | 25.9% | 74.1% |

Definitions: No, number;

* History = History indicative of potentially abnormal hemostasis
