# Supplementary material for: Use and Utility of Hemostatic Screening in Adults Undergoing Elective, Non-Cardiac Surgery
Source: PLoS One. 2015 Dec 1;10(12):e0139139. doi: 10.1371/journal.pone.0139139 (PMC4666643; doi:10.1371/journal.pone.0139139)
Supplement: S6 Table — Table S6A. General demographics, preoperative hemostatic screening tests, patient history variables, and outcomes of interest of plastic surgery patients (n = 15,399). Table S6B. Outcomes stratified by INR values, aPTT values, and platelet count in all plastic surgery patients (n = 15,399). Table S6C. Outcome odds ratios by number of abnormal hemostasis test results in 2,892 plastic surgery patients who underwent all 3 hemostasis tests. Table S6D. Outcome odds ratios by patient “history indicative of potentially abnormal hemostasis” in all plastic surgery patients (n = 15,399). Table S6E. Abnormal screening test odds ratios by patient “history indicative of potentially abnormal hemostasis” in plastic surgery patients screened with all 3 hemostasis tests (n = 2,892). Table S6F. Predictive value of “patient history indicating potentially abnormal coagulation”, abnormal hemostatic test results, both, and neither in plastic surgery patients screened with all 3 hemostatic tests (n = 2,892). (DOCX) [file pone.0139139.s006.docx]

**Table S6A: General demographics, preoperative hemostatic screening tests, patient history variables, and outcomes of interest of plastic surgery patients** (n=15,399)

| **General demographics** | **Frequency** |
| --- | --- |
| Age, years, mean ± SD | 48 ± 13 |
| Female | 15,194 (98.7%) |
| White | 9,447 (68.5%) |
| Admitted from home | 15,376 (99.9%) |
| Partially or fully dependent functional status | 37 (0.2%) |
| ASA | |
| 1 & 2 | 12,501 (81.3%) |
| 3 & 4 | 2,878 (18.7%) |
| 5 | - |
| Prior operation within 30 days | 41 (0.4%) |
| Resident in OR | 3,723 (40.7%) |
| **Preoperative hemostatic screening tests†** | |
| INR | 3,307 (21.5%) |
| aPTT | 3,024 (19.6%) |
| Platelet count | 9,998 (64.9%) |
| All 3 preoperative screening tests were done | 2,892 (18.8%) |
| No preoperative screening tests | 5,308 (34.5%) |
| **Patient history variables indicative of potential bleeding tendency** | |
| Bleeding disorder | 90 (0.6%) |
| Chronic steroid use | 170 (1.1%) |
| Chemotherapy | 238 (1.6%) |
| Radiation therapy | 29 (0.2%) |
| Disseminated cancer | 45 (0.3%) |
| Renal disease | 13 (0.1%) |
| Hepatic disease | 1 (0.01%) |
| History indicative of potentially abnormal hemostasis‡ | 549 (3.6%) |
| **Outcomes of interest** | |
| Perioperative RBC transfusion | 187 (1.2%) |
| Return to the OR | 484 (3.1%) |
| Mortality | 4 (0.03%) |
| Unplanned readmission | 319 (2.1%) |

Definitions: SD, standard deviation or standard difference; ASA = American Association of Anesthesiologists; OR, operating room; INR = International Normalized Ratio; aPTT = activated partial thromboplastin time; RBC = red blood cell;

*Procedures performed, by CPT codes, included, in descending order of frequency, are: 19318, 19357, 19380, 15830, 19325, 19342, 19364, 19340, 19316, 19371.

**Diagnoses included (ICD-9 code), in descending order of frequency, are: 6111.1, 174.9, V51.0, V45.71, 612.1, 174.8, 996.54, 278.1, 612.0, V50.1.

† Number of patients who underwent each of the preoperative hemostatic tests within 90 days prior to surgery.

‡ Patient had one or more of the following risk factors for abnormal haemostasis: history of abnormal bleeding, self-reported family history of bleeding disorders, vitamin K deficiency, currently taking medications that pose a risk for bleeding abnormalities and/or failing to discontinue use of such medications with adequate time for normal hemostasis to be restored, chronic steroid use, chemotherapy and/or radiotherapy for cancer within 90 days prior to surgery, disseminated cancer, renal disease, and/or hepatic disease.

**Table S6B: Outcomes stratified by INR values, aPTT values, and platelet count in all plastic surgery patients** (n=15,399)

| Test and result | No. of patients (%) | No. (%) | | | |
| --- | --- | --- | --- | --- | --- |
|  |  | Perioperative RBC transfusion | Return to the OR | Mortality | Unplanned readmission |
| **INR** | **3,307** |  |  |  |  |
| Normal | 3,241 (98.0%) | 53 (1.6%) | 101 (3.2%) | 2 (0.1%) | 70 (3.2%) |
| Mildly abnormal | 61 (1.8%) | 4 (6.6%) | 6 (9.8%) | 0 (0.0%) | 4 (12.1%) |
| Severely abnormal INR | 5 (0.2%) | 0 (0.0%) | 1 (20.0%) | (0.0%) | 0 (0.0%) |
| All abnormal | 66 (2.0%) | 4 (6.1%) | 7 (10.6%) | 0 (0.0%) | 4 (10.8%) |
| P-value* |  | **0.01** | **<0.01** | 0.98 | **0.02** |
| Sensitivity |  | 0.07 | 0.06 | 0.00 | 0.05 |
| Specificity |  | 0.98 | 0.98 | 0.98 | 0.98 |
| **aPTT** | **3,024** |  |  |  |  |
| Normal | 2,891 (95.6%) | 38 (1.3%) | 92 (3.2%) | 2 (0.1%) | 59 (3.2%) |
| Mildly abnormal | 129 (4.3%) | 6 (4.7%) | 6 (4.7%) | 0 (0.0%) | 5 (5.7%) |
| Severely abnormal | 4 (0.1%) | 0 (0.0%) | 1 (25.0%) | 0 (0.0%) | 1 (25.0%) |
| All abnormal | 133 (4.4%) | 6 (4.5%) | 7 (5.3%) | 0 (0.0%) | 6 (6.5%) |
| P-value* |  | **<0.01** | **0.03** | 0.96 | **0.02** |
| Sensitivity |  | 0.14 | 0.07 | 0.00 | 0.09 |
| Specificity |  | 0.96 | 0.96 | 0.96 | 0.96 |
| **Platelet count** | **9,998** |  |  |  |  |
| Normal | 9,641 (96.4%) | 148 (1.5%) | 341 (3.5%) | 3 (0.03%) | 229 (3.6%) |
| Abnormal low | 257 (2.6%) | 4 (1.6%) | 12 (4.7%) | 0 (0.0%) | 7 (3.8%) |
| Abnormal high | 100 (1.0%) | 2 (2.0%) | 4 (4.0%) | 0 (0.0%) | 3 (5.7%) |
| P-value† |  | **<0.01** | 0.19 | 0.76 | 0.08 |
| Sensitivity‡ |  | 0.03 | 0.03 | 0.00 | 0.03 |
| Sensitivity‡ |  | 0.97 | 0.97 | 0.97 | 0.97 |

Definitions: No, number; aPTT = activated partial thromboplastin time; INR = International Normalized Ratio; RBC = red blood cell; OR = operating room

* All abnormal compared with normal. † Abnormal low platelet count compared with normal platelet count.

‡ Sensitivity and specificity are for abnormal low platelet count only. § Odd ratios and p values that are significant are bolded.

**Table S6C: Outcome odds ratios by number of abnormal hemostasis test results in 2,892 plastic surgery patients who underwent all 3 hemostasis tests**

| Outcome Variables | No. of patients | All 3 tests are within normal range  (n=2,655) | One abnormal test  (n=210) | Odds Ratio* (95% CI) | Two or three abnormal tests  (n=27) | Odds Ratio (95% CI)* | Global P-Value† |
| --- | --- | --- | --- | --- | --- | --- | --- |
| Perioperative RBC transfusion | 43 | 34 (1.3%) | 6 (2.9%) | 2.3 (0.9-5.5) | 3 (11.1%) | **9.6 (2.8-33.5)** | **<0.001** |
| Return to the OR | 95 | 82 (3.1%) | 10 (4.8%) | 1.6 (0.8-3.1) | 3 (11.1%) | **3.9 (1.2-13.3)** | **0.03** |
| Mortality | 2 | 2 (0.1%) | 0 (0.0%) | N/C | 0 (0.0%) | N/C | 0.91 |
| Unplanned readmission | 63 | 56 (3.3%) | 5 (3.6%) | 1.1 (0.4-2.8) | 2 (15.4%) | **5.4 (1.2-24.9)** | 0.05 |

Definitions: No, number; CI = confidence interval; OR = operating room; RBC = red blood cell; N/C, not calculable

* Odd ratios are relative to all three tests within normal range.

† Pearson's chi-square test used to compare differences in outcomes across all groups.

‡ Odd ratios and p values that are significant are bolded.

**Table S6D: Outcome odds ratios by patient “history indicative of potentially abnormal hemostasis” in all plastic surgery patients (**n=15,399)

| Outcome Variables | No. of patients | No history*  (n=14,850) | History*  (n=549) | Odds Ratio  (95% CI) | P-Value | Sensitivity | Specificity |
| --- | --- | --- | --- | --- | --- | --- | --- |
| Perioperative RBC transfusion | 187 | 171 (1.2%) | 16 (2.9%) | **2.6 (1.5-4.3)** | **<0.001** | 0.09 | 0.97 |
| Return to the OR | 484 | 461 (3.1%) | 23 (4.2%) | 1.4 (0.9-2.1) | 0.15 | 0.05 | 0.96 |
| Mortality | 4 | 4 (0.03%) | 0 (0.0%) | N/C | 0.70 | 0.00 | 0.96 |
| Unplanned readmission | 319 | 291 (3.0%) | 28 (7.9%) | **2.8 (1.9-4.2)** | **<0.001** | 0.09 | 0.97 |

Definitions: No, number; CI = confidence interval; RBC = red blood cell; OR = operating room; N?C, not calculable

* History = History indicative of potentially abnormal hemostasis

† Odd ratios and p values that are significant are bolded.

**Table S6E: Abnormal screening test odds ratios by patient “history indicative of potentially abnormal hemostasis” in plastic surgery patients screened with all 3 hemostasis tests** (n=2,892)

| Test Findings | No. of patients | No history*  (n=134) | History*  (n=2,758) | Odds Ratio  (95% CI) | P-Value |
| --- | --- | --- | --- | --- | --- |
| Mildly abnormal INR | 40 | 33 | 7 | **4.6 (2.0-10.5)** | **<0.001** |
| Severely abnormal INR | 3 | 3 | 0 | N/C | 0.70 |
| All abnormal INR | 43 | 36 | 7 | **4.2 (1.8-9.5)** | **<0.001** |
| Mildly abnormal aPTT | 125 | 114 | 11 | **2.1 (1.1-4.0)** | **0.02** |
| Severely abnormal aPTT | 4 | 3 | 1 | 6.9 (0.7-66.8) | 0.05 |
| All abnormal aPTT | 129 | 117 | 12 | **2.2 (1.2-4.1)** | **<0.01** |
| Abnormal low platelet count | 74 | 69 | 5 | 1.5 (0.6-3.8) | 0.38 |
| Abnormal high platelet count | 19 | 18 | 1 | 1.1 (0.2-8.6) | 0.90 |

Definition: No, number; aPTT = activated partial thromboplastin time; CI = confidence interval; INR = International Normalized Ratio; OR = operating room; RBC = red blood cell; N/C, not calculable

* History = History indicative of potentially abnormal hemostasis

† Odd ratios and p values that are significant are bolded.

**Table S6F: Predictive value of “patient history indicating potentially abnormal coagulation”, abnormal hemostatic test results, both, and neither in plastic surgery patients screened with all 3 hemostatic tests** (n=2,892)

| Outcome Variables | No. of patients | History* | >1 abnormal test | With history* and/or >1 abnormal test | Without history* and no abnormal coagulation tests |
| --- | --- | --- | --- | --- | --- |
| No. of patients |  | 134 | 237 | 350 | 2,542 |
| Perioperative RBC transfusion | 43 | 9.3% | 20.9% | 30.2% | 69.8% |
| Return to the OR | 95 | 5.3% | 13.7% | 16.8% | 83.2% |
| Mortality | 2 | 0.0% | 0.0% | 0.0% | 100.0% |
| Unplanned readmission | 63 | 9.5% | 11.1% | 19.1% | 63.6% |

Definitions: No, number.

* History = History indicative of potentially abnormal hemostasis
