# Supplementary material for: Use and Utility of Hemostatic Screening in Adults Undergoing Elective, Non-Cardiac Surgery
Source: PLoS One. 2015 Dec 1;10(12):e0139139. doi: 10.1371/journal.pone.0139139 (PMC4666643; doi:10.1371/journal.pone.0139139)
Supplement: S7 Table — Table S7A. General demographics, preoperative hemostatic screening tests, patient history variables, and outcomes of interest of thoracic surgery patients (n = 7,758). Table S7B. Outcomes stratified by INR values, aPTT values, and platelet count in all thoracic surgery patients (n = 7,758). Table S7C. Outcome odds ratios by number of abnormal hemostasis test results in 5,139 thoracic surgery patients who underwent all 3 hemostasis tests. Table S7D. Outcome odds ratios by number of abnormal hemostasis test results in 5,139 thoracic surgery patients who underwent all 3 hemostasis tests. Table S7E. Abnormal screening test odds ratios by patient “history indicative of potentially abnormal hemostasis” in thoracic surgery patients screened with all 3 hemostasis tests (n = 5,139). Table S7F. Predictive values, stratified by the presence or absence of a positive history for potentially abnormal hemostasis, and pre-operative screening in all patients (n = 728,135) who underwent all 3 hematologic tests prior to surgery. (DOCX) [file pone.0139139.s007.docx]

**Table S7A: General demographics, preoperative hemostatic screening tests, patient history variables, and outcomes of interest of thoracic surgery patients** (n=7,758)

| **General demographics** | **Frequency** |
| --- | --- |
| Age, years, mean ± SD | 65 ± 12 |
| Female | 4,063 (52.4%) |
| White | 6,059 (81.1%) |
| Admitted from home | 7,647 (98.6%) |
| Partially or fully dependent functional status | 175 (2.3%) |
| ASA | |
| 1 & 2 | 1,649 (21.3%) |
| 3 & 4 | 6,097 (78.7%) |
| 5 | 3 (0.04%) |
| Prior operation within 30 days | 158 (3.5%) |
| Resident in OR | 2,299 (51.0%) |
| **Preoperative hemostatic screening tests†** | |
| INR | 5,980 (77.1%) |
| aPTT | 5,200 (67.0%) |
| Platelet count | 7,530 (97.1%) |
| All 3 preoperative screening tests were done | 5,139 (66.2%) |
| No preoperative screening tests | 177 (2.3%) |
| **Patient history variables indicative of potential bleeding tendency** | |
| Bleeding disorder | 291 (3.8%) |
| Chronic steroid use | 377 (4.9%) |
| Chemotherapy | 162 (2.1%) |
| Radiation therapy | 99 (1.3%) |
| Disseminated cancer | 778 (10.0%) |
| Renal disease | 59 (0.8%) |
| Hepatic disease | 16 (0.2%) |
| History indicative of potentially abnormal hemostasis‡ | 1,539 (19.8%) |
| **Outcomes of interest** | |
| Perioperative RBC transfusion | 335 (4.3%) |
| Return to the OR | 390 (5.0%) |
| Mortality | 135 (1.7%) |
| Unplanned readmission | 482 (6.2%) |

Definitions: SD, standard deviation or standard difference; ASA = American Association of Anesthesiologists; OR, operating room; INR = International Normalized Ratio; aPTT = activated partial thromboplastin time; RBC = red blood cell;

*Procedures performed, by CPT codes, included, in descending order of frequency, are: 32480, 32663, 32657, 39400, 32666, 32650, 32500, 43117, 32505, 32651.

**Diagnoses included (ICD-9 code), in descending order of frequency, are: 162.3, 162.5, 197.0, 162.9, 518.89, 786.6, 515, 511.9, 510.9, 785.6.

† Number of patients who underwent each of the preoperative hemostatic tests within 90 days prior to surgery.

‡ Patient had one or more of the following risk factors for abnormal haemostasis: history of abnormal bleeding, self-reported family history of bleeding disorders, vitamin K deficiency, currently taking medications that pose a risk for bleeding abnormalities and/or failing to discontinue use of such medications with adequate time for normal hemostasis to be restored, chronic steroid use, chemotherapy and/or radiotherapy for cancer within 90 days prior to surgery, disseminated cancer, renal disease, and/or hepatic disease.

**Table S7B: Outcomes stratified by INR values, aPTT values, and platelet count in all thoracic surgery patients** (n=7,758)

| Test and result | No. of patients (%) | No. (%) | | | |
| --- | --- | --- | --- | --- | --- |
|  |  | Perioperative RBC transfusion | Return to the OR | Mortality | Unplanned readmission |
| **INR** | **5,980** |  |  |  |  |
| Normal | 5,650 (94.5%) | 236 (4.2%) | 264 (4.7%) | 89 (1.6%) | 357 (10.3%) |
| Mildly abnormal | 322 (5.4%) | 31 (9.6%) | 17 (5.3%) | 24 (7.5%) | 20 (11.6%) |
| Severely abnormal INR | 8 (0.1%) | 0 (0.0%) | 1 (12.5%) | 1 (12.5%) | 1 (20.0%) |
| All abnormal | 330 (5.5%) | 31 (9.4%) | 18 (5.5%) | 25 (7.6%) | 21 (11.8%) |
| P-value* |  | **<0.001** | 0.51 | **<0.001** | 0.51 |
| Sensitivity |  | 0.11 | 0.06 | 0.22 | 0.06 |
| Specificity |  | 0.95 | 0.95 | 0.95 | 0.95 |
| **aPTT** | **5,200** |  |  |  |  |
| Normal | 4,734 (91.0%) | 215 (4.5%) | 231 (4.9%) | 79 (1.7%) | 289 (10.4%) |
| Mildly abnormal | 448 (8.6%) | 22 (4.9%) | 23 (5.3%) | 19 (4.2%) | 30 (11.5%) |
| Severely abnormal | 18 (0.4%) | 2 (11.1%) | 2 (11.1%) | 3 (16.7%) | 3 (30.0%) |
| All abnormal | 466 (9.0%) | 24 (5.2%) | 25 (5.4%) | 22 (4.7%) | 33 (12.2%) |
| P-value* |  | 0.55 | 0.64 | **<0.001** | 0.35 |
| Sensitivity |  | 0.10 | 0.10 | 0.22 | 0.10 |
| Specificity |  | 0.91 | 0.91 | 0.91 | 0.91 |
| **Platelet count** | **7,530** |  |  |  |  |
| Normal | 6,724 (89.3%) | 257 (3.8%) | 334 (5.0%) | 106 (1.6%) | 419 (10.2%) |
| Abnormal low | 574 (7.6%) | 44 (7.7%) | 29 (5.1%) | 17 (3.0%) | 31 (8.3%) |
| Abnormal high | 232 (3.1%) | 23 (9.9%) | 19 (8.2%) | 11 (4.7%) | 16 (13.1%) |
| P-value† |  | **<0.001** | 0.98 | **0.03** | 0.23 |
| Sensitivity‡ |  | 0.14 | 0.08 | 0.13 | 0.07 |
| Sensitivity‡ |  | 0.93 | 0.92 | 0.92 | 0.92 |

Definitions: No, number; aPTT = activated partial thromboplastin time; INR = International Normalized Ratio; RBC = red blood cell; OR = operating room

* All abnormal compared with normal. † Abnormal low platelet count compared with normal platelet count.

‡ Sensitivity and specificity are for abnormal low platelet count only. § Odd ratios and p values that are significant are bolded.

**Table S7C: Outcome odds ratios by number of abnormal hemostasis test results in 5,139 thoracic surgery patients who underwent all 3 hemostasis tests**

| Outcome Variables | No. of patients | All 3 tests are within normal range  (n=4,072) | One abnormal test  (n=868) | Odds Ratio* (95% CI) | Two or three abnormal tests  (n=199) | Odds Ratio (95% CI)* | Global P-Value† |
| --- | --- | --- | --- | --- | --- | --- | --- |
| Perioperative RBC transfusion | 235 | 157 (3.9%) | 62 (7.1%) | **1.9 (1.4-2.6)** | 16 (8.0%) | **2.2 (1.3-3.7)** | **<0.001** |
| Return to the OR | 251 | 199 (4.9%) | 40 (4.6%) | 0.9 (0.7-1.3) | 12 (6.0%) | 1.2 (0.7-2.3) | 0.70 |
| Mortality | 100 | 54 (1.3%) | 31 (3.6%) | **2.8 (1.8-4.3)** | 15 (7.5%) | **6.1 (3.4-11.0)** | **<0.001** |
| Unplanned readmission | 316 | 251 (10.4%) | 50 (9.8%) | 0.9 (0.7-1.3) | 15 (13.8%) | 1.4 (0.8-2.4) | 0.46 |

Definitions: No, number; CI = confidence interval; OR = operating room; RBC = red blood cell

* Odd ratios are relative to all three tests within normal range.

† Pearson's chi-square test used to compare differences in outcomes across all groups.

‡ Odd ratios and p values that are significant are bolded.

**Table S7D: Outcome odds ratios by patient “history indicative of potentially abnormal hemostasis” in all thoracic surgery patients** (n=7,758)

| Outcome Variables | No. of patients | No history*  (n=6,219) | History*  (n=1,539) | Odds Ratio  (95% CI) | P-Value | Sensitivity | Specificity |
| --- | --- | --- | --- | --- | --- | --- | --- |
| Perioperative RBC transfusion | 335 | 240 (3.9%) | 96 (6.2%) | **1.6 (1.3-2.1)** | **<0.001** | 0.28 | 0.81 |
| Return to the OR | 390 | 320 (5.2%) | 70 (4.6%) | 0.9 (0.7-1.1) | 0.34 | 0.18 | 0.80 |
| Mortality | 135 | 80 (1.3%) | 55 (3.6%) | **2.8 (2.0-4.0)** | **<0.001** | 0.41 | 0.81 |
| Unplanned readmission | 482 | 379 (10.1%) | 103 (10.9%) | 1.1 (0.9-1.4) | 0.47 | 0.21 | 0.80 |

Definitions: No, number; CI = confidence interval; RBC = red blood cell; OR = operating room

* History = History indicative of potentially abnormal hemostasis

† Odd ratios and p values that are significant are bolded.

**Table S7E: Abnormal screening test odds ratios by patient “history indicative of potentially abnormal hemostasis” in thoracic surgery patients screened with all 3 hemostasis tests** (n=5,139)

| Test Findings | No. of patients | No history*  (n=4,115) | History*  (n=1,024) | Odds Ratio  (95% CI) | P-Value |
| --- | --- | --- | --- | --- | --- |
| Mildly abnormal INR | 264 | 167 | 97 | **2.5 (1.9-3.2)** | **<0.001** |
| Severely abnormal INR | 5 | 3 | 2 | 2.7 (0.4-16.1) | 0.26 |
| All abnormal INR | 269 | 170 | 99 | **2.5 (1.9-3.2)** | **<0.001** |
| Mildly abnormal aPTT | 441 | 323 | 118 | **1.5 (1.2-1.9)** | **<0.001** |
| Severely abnormal aPTT | 18 | 10 | 8 | **3.2 (1.3-8.2)** | **0.01** |
| All abnormal aPTT | 459 | 333 | 126 | **1.6 (1.3-2.0)** | **<0.001** |
| Abnormal low platelet count | 403 | 263 | 140 | **2.3 (1.9-2.9)** | **<0.001** |
| Abnormal high platelet count | 162 | 138 | 24 | 0.7 (0.4-1.1) | 0.10 |

Definitions: No, number; aPTT = activated partial thromboplastin time; CI = confidence interval; INR = International Normalized Ratio; OR = operating room; RBC = red blood cell

* History = History indicative of potentially abnormal hemostasis

† Odd ratios and p values that are significant are bolded.

**Table S7F: Predictive value of “patient history indicating potentially abnormal coagulation”, abnormal hemostatic test results, both, and neither in thoracic surgery patients screened with all 3 hemostatic tests** (n=5,139)

| Outcome Variables | No. of patients | History* | >1 abnormal test | With history* and/or >1 abnormal test | Without history* and no abnormal coagulation tests |
| --- | --- | --- | --- | --- | --- |
| No. of patients |  | 1,024 | 1,067 | 1,795 | 3,344 |
| Perioperative RBC transfusion | 235 | 31.5% | 33.2% | 51.1% | 48.9% |
| Return to the OR | 251 | 18.7% | 20.7% | 32.7% | 67.3% |
| Mortality | 100 | 43.0% | 46.0% | 64.0% | 36.0% |
| Unplanned readmission | 316 | 21.8% | 20.6% | 36.4% | 63.6% |

Definitions: No, number.

* History = History indicative of potentially abnormal hemostasis
