# Supplementary material for: Use and Utility of Hemostatic Screening in Adults Undergoing Elective, Non-Cardiac Surgery
Source: PLoS One. 2015 Dec 1;10(12):e0139139. doi: 10.1371/journal.pone.0139139 (PMC4666643; doi:10.1371/journal.pone.0139139)
Supplement: S8 Table — Table S8A. General demographics, preoperative hemostatic screening tests, patient history variables, and outcomes of interest of urological surgery patients (n = 34,258). Table S8B. Outcomes stratified by INR values, aPTT values, and platelet count in all urological surgery patients (n = 34,258). Table S8C. Outcome odds ratios by number of abnormal hemostasis test results in 14,254 urological surgery patients who underwent all 3 hemostasis tests. Table S9D. Outcome odds ratios by patient “history indicative of potentially abnormal hemostasis” in all urological surgery patients (n = 34,258). Table S8E. Abnormal screening test odds ratios by patient “history indicative of potentially abnormal hemostasis” in urological surgery patients screened with all 3 hemostasis tests (n = 14,254). Table S8F. Predictive value of “patient history indicating potentially abnormal coagulation”, abnormal hemostatic test results, both, and neither in urological surgery patients screened with all 3 hemostatic tests (n = 14,254). (DOCX) [file pone.0139139.s008.docx]

**Table S8A: General demographics, preoperative hemostatic screening tests, patient history variables, and outcomes of interest of urological surgery patients** (n=34,258)

| **General demographics** | **Frequency** |
| --- | --- |
| Age, years, mean ± SD | 65 ± 11 |
| Female | 6,234 (18.2%) |
| White | 25,659 (79.2%) |
| Admitted from home | 33,924 (99.0%) |
| Partially or fully dependent functional status | 665 (2.0%) |
| ASA | |
| 1 & 2 | 18.852 (55.1%) |
| 3 & 4 | 15,360 (44.9%) |
| 5 | 1 (0.0%) |
| Prior operation within 30 days | 150 (0.8%) |
| Resident in OR | 8,344 (41.1%) |
| **Preoperative hemostatic screening tests†** | |
| INR | 16,791 (49.0%) |
| aPTT | 14,753 (43.1%) |
| Platelet count | 30,285 (88.4%) |
| All 3 preoperative screening tests were done | 14,254 (41.6%) |
| No preoperative screening tests | 3,628 (10.6%) |
| **Patient history variables indicative of potential bleeding tendency** | |
| Bleeding disorder | 756 (2.2%) |
| Chronic steroid use | 677 (2.0%) |
| Chemotherapy | 107 (0.3%) |
| Radiation therapy | 42 (0.1%) |
| Disseminated cancer | 409 (1.2%) |
| Renal disease | 189 (0.6%) |
| Hepatic disease | 21 (0.1%) |
| History indicative of potentially abnormal hemostasis‡ | 2025 (5.9%) |
| **Outcomes of interest** | |
| Perioperative RBC transfusion | 773 (2.3%) |
| Return to the OR | 613 (1.8%) |
| Mortality | 105 (0.3%) |
| Unplanned readmission | 1,083 (3.2%) |

Definitions: SD, standard deviation or standard difference; ASA = American Association of Anesthesiologists; OR, operating room; INR = International Normalized Ratio; aPTT = activated partial thromboplastin time; RBC = red blood cell;

*Procedures performed, by CPT codes, included, in descending order of frequency, are: 55866, 52601, 52234, 52648, 57288, 52235, 50543, 52240, 50545, 50240.

**Diagnoses included (ICD-9 code), in descending order of frequency, are: 185, 600.01, 189.0, 188.9, 625.6, 188.8, 593.9, 603.9, 600.00, 188.2.

† Number of patients who underwent each of the preoperative hemostatic tests within 90 days prior to surgery.

‡ Patient had one or more of the following risk factors for abnormal haemostasis: history of abnormal bleeding, self-reported family history of bleeding disorders, vitamin K deficiency, currently taking medications that pose a risk for bleeding abnormalities and/or failing to discontinue use of such medications with adequate time for normal hemostasis to be restored, chronic steroid use, chemotherapy and/or radiotherapy for cancer within 90 days prior to surgery, disseminated cancer, renal disease, and/or hepatic disease.

**Table S8B: Outcomes stratified by INR values, aPTT values, and platelet count in all urological surgery patients** (n=34,258)

| Test and result | No. of patients (%) | No. (%) | | | |
| --- | --- | --- | --- | --- | --- |
|  |  | Perioperative RBC transfusion | Return to the OR | Mortality | Unplanned readmission |
| **INR** | **16,791** |  |  |  |  |
| Normal | 15,895 (94.7%) | 422 (2.7%) | 281 (1.8%) | 55 (0.4%) | 530 (5.4%) |
| Mildly abnormal | 843 (5.0%) | 53 (6.3%) | 29 (3.4%) | 18 (2.1%) | 50 (10.7%) |
| Severely abnormal INR | 53 (0.3%) | 3 (5.7%) | 2 (3.8%) | 0 (0.0%) | 3 (12.5%) |
| All abnormal | 896 (5.3%) | 56 (6.3%) | 31 (3.5%) | 18 (2.0%) | 53 (10.8%) |
| P-value* |  | **<0.001** | **<0.001** | **<0.001** | **<0.001** |
| Sensitivity |  | 0.12 | 0.10 | 0.25 | 0.09 |
| Specificity |  | 0.95 | 0.95 | 0.95 | 0.95 |
| **aPTT** | **14,753** |  |  |  |  |
| Normal | 13,817 (93.7%) | 381 (2.8%) | 241 (1.7%) | 49 (0.4%) | 424 (5.1%) |
| Mildly abnormal | 898 (6.1%) | 43 (4.8%) | 19 (2.1%) | 7 (0.8%) | 64 (11.2%) |
| Severely abnormal | 38 (0.3%) | 2 (5.3%) | 3 (7.9%) | 1 (2.6%) | 4 (22.2%) |
| All abnormal | 936 (6.3%) | 45 (4.8%) | 22 (2.4%) | 8 (0.9%) | 68 (11.5%) |
| P-value* |  | **<0.001** | 0.18 | **0.02** | **<0.001** |
| Sensitivity |  | 0.11 | 0.08 | 0.14 | 0.14 |
| Specificity |  | 0.94 | 0.94 | 0.94 | 0.94 |
| **Platelet count** | **30,285** |  |  |  |  |
| Normal | 27,594 (91.1%) | 632 (2.3%) | 453 (1.6%) | 85 (0.3%) | 868 (5.1%) |
| Abnormal low | 2,433 (8.0%) | 81 (3.3%) | 79 (3.3%) | 13 (0.5%) | 125 (7.9%) |
| Abnormal high | 258 (0.9%) | 31 (12.0%) | 8 (3.1%) | 1 (0.4%) | 13 (8.5%) |
| P-value† |  | **<0.01** | **<0.001** | 0.06 | **<0.001** |
| Sensitivity‡ |  | 0.11 | 0.15 | 0.13 | 0.12 |
| Sensitivity‡ |  | 0.92 | 0.92 | 0.92 | 0.92 |

Definitions: No, number; aPTT = activated partial thromboplastin time; INR = International Normalized Ratio; RBC = red blood cell; OR = operating room

* All abnormal compared with normal. † Abnormal low platelet count compared with normal platelet count.

‡ Sensitivity and specificity are for abnormal low platelet count only. § Odd ratios and p values that are significant are bolded.

**Table S8C: Outcome odds ratios by number of abnormal hemostasis test results in 14,254 urological surgery patients who underwent all 3 hemostasis tests**

| Outcome Variables | No. of patients | All 3 tests are within normal range  (n=11,898) | One abnormal test  (n=1,981) | Odds Ratio* (95% CI) | Two or three abnormal tests  (n=375) | Odds Ratio (95% CI)* | Global P-Value† |
| --- | --- | --- | --- | --- | --- | --- | --- |
| Perioperative RBC transfusion | 415 | 298 (2.5%) | 91 (4.6%) | **1.9 (1.5-2.4)** | 26 (6.3%) | **2.9 (1.9-4.4)** | **<0.001** |
| Return to the OR | 252 | 189 (1.6%) | 49 (2.5%) | **1.6 (1.1-2.2)** | 14 (3.7%) | **2.4 (1.4-4.2)** | **<0.001** |
| Mortality | 55 | 30 (0.3%) | 18 (0.9%) | **3.6 (2.0-6.5)** | 7 (1.9%) | **7.5 (3.3-17.2)** | **<0.001** |
| Unplanned readmission | 479 | 340 (4.7%) | 110 (9.2%) | **2.0 (1.6-2.5)** | 29 (12.4%) | **2.8 (1.9-4.3)** | **<0.001** |

Definitions: No, number; CI = confidence interval; OR = operating room; RBC = red blood cell

* Odd ratios are relative to all three tests within normal range.

† Pearson's chi-square test used to compare differences in outcomes across all groups.

‡ Odd ratios and p values that are significant are bolded.

**Table S8D: Outcome odds ratios by patient “history indicative of potentially abnormal hemostasis” in all urological surgery patients** (n=34,258)

| Outcome Variables | No. of patients | No history*  (n=32,233) | History*  (n=2,025) | Odds Ratio  (95% CI) | P-Value | Sensitivity | Specificity |
| --- | --- | --- | --- | --- | --- | --- | --- |
| Perioperative RBC transfusion | 773 | 660 (2.1%) | 113 (5.6%) | **2.8 (2.3-3.5)** | **<0.001** | 0.15 | 0.94 |
| Return to the OR | 613 | 555 (1.7%) | 58 (2.9%) | **1.7 (1.3-2.2)** | **<0.001** | 0.09 | 0.94 |
| Mortality | 105 | 71 (0.2%) | 34 (1.7%) | **7.7 (5.1-11.7)** | **<0.001** | 0.32 | 0.94 |
| Unplanned readmission | 1,083 | 926 (4.6%) | 157 (11.8%) | **2.8 (2.3-3.3)** | **<0.001** | 0.15 | 0.94 |

Definitions: No, number; CI = confidence interval; RBC = red blood cell; OR = operating room

* History = History indicative of potentially abnormal hemostasis

† Odd ratios and p values that are significant are bolded.

**Table S8E: Abnormal screening test odds ratios by patient “history indicative of potentially abnormal hemostasis” in urological surgery patients screened with all 3 hemostasis tests** (n=14,254)

| Test Findings | No. of patients | No history*  (n=13,227) | History*  (n=1,027) | Odds Ratio  (95% CI) | P-Value |
| --- | --- | --- | --- | --- | --- |
| Mildly abnormal INR | 584 | 437 | 147 | **4.9 (4.0-6.0)** | **<0.001** |
| Severely abnormal INR | 31 | 22 | 9 | **5.3 (2.4-11.6)** | **<0.001** |
| All abnormal INR | 615 | 459 | 156 | **5.0 (4.1-6.1)** | **<0.001** |
| Mildly abnormal aPTT | 853 | 697 | 156 | **3.2 (2.7-3.9)** | **<0.001** |
| Severely abnormal aPTT | 35 | 25 | 10 | **5.2 (2.5-10.8)** | **<0.001** |
| All abnormal aPTT | 888 | 722 | 166 | **3.3 (2.8-4.0)** | **<0.001** |
| Abnormal low platelet count | 1,141 | 947 | 194 | **3.0 (2.5-3.6)** | **<0.001** |
| Abnormal high platelet count | 135 | 110 | 25 | **3.0 (1.9-4.6)** | **<0.001** |

aPTT = activated partial thromboplastin time; CI = confidence interval; INR = International Normalized Ratio; OR = operating room; RBC = red blood cell

* History = History indicative of potentially abnormal hemostasis

† Odd ratios and p values that are significant are bolded.

**Table S8F: Predictive value of “patient history indicating potentially abnormal coagulation”, abnormal hemostatic test results, both, and neither in urological surgery patients screened with all 3 hemostatic tests** (n=14,254)

| Outcome Variables | No. of patients | History* | >1 abnormal test | With history* and/or >1 abnormal test | Without history* and no abnormal coagulation tests |
| --- | --- | --- | --- | --- | --- |
| No. of patients |  | 1,027 | 2,356 | 2,979 | 11,275 |
| Perioperative RBC transfusion | 415 | 17.1% | 28.2% | 36.6% | 63.4% |
| Return to the OR | 252 | 11.1% | 25.0% | 32.1% | 67.9% |
| Mortality | 55 | 36.4% | 45.5% | 81.8% | 18.2% |
| Unplanned readmission | 479 | 18.4% | 29.0% | 39.0% | 61.0% |

Definitions: No, number.

* History = History indicative of potentially abnormal hemostasis
