# Supplementary material for: Use and Utility of Hemostatic Screening in Adults Undergoing Elective, Non-Cardiac Surgery
Source: PLoS One. 2015 Dec 1;10(12):e0139139. doi: 10.1371/journal.pone.0139139 (PMC4666643; doi:10.1371/journal.pone.0139139)
Supplement: S9 Table — Table S9A. General demographics, preoperative hemostatic screening tests, patient history variables, and outcomes of interest of vascular surgery patients (n = 83,101). Table S9B. Outcomes stratified by INR values, aPTT values, and platelet count in all vascular surgery patients (n = 83,101). Table S9C. Outcome odds ratios by number of abnormal hemostasis test results in 50,839 vascular surgery patients who underwent all 3 hemostasis tests. Table S9D. Outcome odds ratios by patient “history indicative of potentially abnormal hemostasis” in all vascular surgery patients (n = 83,101). Table S9E. Abnormal screening test odds ratios by patient “history indicative of potentially abnormal hemostasis” in vascular surgery patients screened with all 3 hemostasis tests (n = 50,839). Table S9F. Predictive value of “patient history indicating potentially abnormal coagulation”, abnormal hemostatic test results, both, and neither in vascular surgery patients screened with all 3 hemostatic tests (n = 50,839). (DOCX) [file pone.0139139.s009.docx]

**Table S9A: General demographics, preoperative hemostatic screening tests, patient history variables, and outcomes of interest of vascular surgery patients** (n=83,101)

| **General demographics** | **Frequency** |
| --- | --- |
| Age, years, mean ± SD | 69 ± 12 |
| Female | 32,916 (39.6%) |
| White | 67,760 (84.2%) |
| Admitted from home | 79,286 (95.4%) |
| Partially or fully dependent functional status | 6,376 (7.7%) |
| ASA | |
| 1 & 2 | 12,043 (14.7%) |
| 3 & 4 | 69,911 (85.3%) |
| 5 | 29 (0.04%) |
| Prior operation within 30 days | 1,745 (2.8%) |
| Resident in OR | 39,203 (58.7%) |
| **Preoperative hemostatic screening tests†** | |
| INR | 58,812 (70.8%) |
| aPTT | 51,155 (62.8%) |
| Platelet count | 76,938 (92.6%) |
| All 3 preoperative screening tests were done | 50,839 (61.2%) |
| No preoperative screening tests | 5,155 (6.2%) |
| **Patient history variables indicative of potential bleeding tendency** | |
| Bleeding disorder | 14,612 (17.6%) |
| Chronic steroid use | 2,431 (2.9%) |
| Chemotherapy | 172 (0.2%) |
| Radiation therapy | 91 (0.1%) |
| Disseminated cancer | 258 (0.3%) |
| Renal disease | 2,422 (2.9%) |
| Hepatic disease | 128 (0.2%) |
| History indicative of potentially abnormal hemostasis‡ | 18,630 (22.4%) |
| **Outcomes of interest** | |
| Perioperative RBC transfusion | 5,519 (6.6%) |
| Return to the OR | 4,855 (5.8%) |
| Mortality | 971 (1.2%) |
| Unplanned readmission | 2,484 (3.0%) |

Definitions: SD, standard deviation or standard difference; ASA = American Association of Anesthesiologists; OR, operating room; INR = International Normalized Ratio; aPTT = activated partial thromboplastin time; RBC = red blood cell;

*Procedures performed, by CPT codes, included, in descending order of frequency, are: 35301, 34802, 36475, 35556, 35656, 34803, 27880, 35566, 27590, 37765.

**Diagnoses included (ICD-9 code), in descending order of frequency, are: 433.10, 441.4, 454.8, 440.21, 440.24, 440.23, 443.9, 996.74, 459.81.

† Number of patients who underwent each of the preoperative hemostatic tests within 90 days prior to surgery.

‡ Patient had one or more of the following risk factors for abnormal haemostasis: history of abnormal bleeding, self-reported family history of bleeding disorders, vitamin K deficiency, currently taking medications that pose a risk for bleeding abnormalities and/or failing to discontinue use of such medications with adequate time for normal hemostasis to be restored, chronic steroid use, chemotherapy and/or radiotherapy for cancer within 90 days prior to surgery, disseminated cancer, renal disease, and/or hepatic disease.

**Table S9B: Outcomes stratified by INR values, aPTT values, and platelet count in all vascular surgery patients** (n=83,101)

| Test and result | No. of patients (%) | No. (%) | | | |
| --- | --- | --- | --- | --- | --- |
|  |  | Perioperative RBC transfusion | Return to the OR | Mortality | Unplanned readmission |
| **INR** | **58,812** |  |  |  |  |
| Normal | 52,394 (89.1%) | 3,823 (7.3%) | 3,175 (6.1%) | 549 (1.1%) | 1,644 (9.7%) |
| Mildly abnormal | 6,193 (10.5%) | 899 (14.5%) | 649 (10.5%) | 271 (4.4%) | 279 (15.2%) |
| Severely abnormal INR | 225 (0.4%) | 25 (11.1%) | 20 (8.9%) | 4 (1.8%) | 8 (12.9%) |
| All abnormal | 6,418 (10.9%) | 924 (14.4%) | 669 (10.4%) | 275 (4.3%) | 287 (15.1%) |
| P-value* |  | **<0.001** | **<0.001** | **<0.001** | **<0.001** |
| Sensitivity |  | 0.19 | 0.17 | 0.33 | 0.15 |
| Specificity |  | 0.90 | 0.90 | 0.89 | 0.90 |
| **aPTT** | **52,155** |  |  |  |  |
| Normal | 43,631 (83.7%) | 3,044 (7.0%) | 2,544 (5.8%) | 461 (1.1%) | 1,271 (9.6%) |
| Mildly abnormal | 6,861 (13.2%) | 983 (14.3%) | 683 (10.0%) | 214 (3.1%) | 279 (13.6%) |
| Severely abnormal | 1,663 (3.2%) | 275 (16.5%) | 227 (13.7%) | 52 (3.1%) | 90 (16.8%) |
| All abnormal | 8,524 (16.3%) | 1,258 (14.8%) | 910 (10.7%) | 266 (3.1%) | 369 (14.2%) |
| P-value* |  | **<0.001** | **<0.001** | **<0.001** | **<0.001** |
| Sensitivity |  | 0.29 | 0.26 | 0.34 | 0.23 |
| Specificity |  | 0.85 | 0.84 | 0.84 | 0.84 |
| **Platelet count** | **76,938** |  |  |  |  |
| Normal | 67,928 (88.3%) | 4,390 (6.5%) | 3,933 (5.8%) | 749 (1.1%) | 2044 (9.2%) |
| Abnormal low | 7,284 (9.5%) | 712 (9.8%) | 408 (5.6%) | 144 (2.0%) | 274 (10.4%) |
| Abnormal high | 1,726 (2.2%) | 302 (17.5%) | 235 (13.6%) | 55 (3.2%) | 78 (18.0%) |
| P-value† |  | **<0.001** | 0.19 | **<0.001** | 0.08 |
| Sensitivity‡ |  | 0.13 | 0.09 | 0.15 | 0.11 |
| Sensitivity‡ |  | 0.91 | 0.91 | 0.91 | 0.90 |

Definitions: No, number; aPTT = activated partial thromboplastin time; INR = International Normalized Ratio; RBC = red blood cell; OR = operating room

* All abnormal compared with normal. † Abnormal low platelet count compared with normal platelet count.

‡ Sensitivity and specificity are for abnormal low platelet count only. § Odd ratios and p values that are significant are bolded.

**Table S9C: Outcome odds ratios by number of abnormal hemostasis test results in 50,839 vascular surgery patients who underwent all 3 hemostasis tests**

| Outcome Variables | No. of patients | All 3 tests are within normal range  (n=35,778) | One abnormal test  (n=10,967) | Odds Ratio* (95% CI) | Two or three abnormal tests  (n=4,094) | Odds Ratio (95% CI)* | Global P-Value† |
| --- | --- | --- | --- | --- | --- | --- | --- |
| Perioperative RBC transfusion | 4,231 | 2,201 (6.2%) | 1,322 (12.1%) | **2.1 (1.9-2.3)** | 708 (17.3%) | **3.2 (2.9-3.5)** | **<0.001** |
| Return to the OR | 3,394 | 1,986 (5.6%) | 925 (8.4%) | **1.6 (1.4-1.7)** | 483 (11.8%) | **2.3 (2.0-2.5)** | **<0.001** |
| Mortality | 719 | 286 (0.8%) | 240 (2.2%) | **2.8 (2.3-3.3)** | 193 (4.7%) | **6.1 (5.1-7.4)** | **<0.001** |
| Unplanned readmission | 1,619 | 989 (9.1%) | 434 (12.1%) | **1.5 (1.3-1.6)** | 196 (16.5%) | **2.0 (1.7-2.3)** | **<0.001** |

Definitions: No, number; CI = confidence interval; OR = operating room; RBC = red blood cell

* Odd ratios are relative to all three tests within normal range.

† Pearson's chi-square test used to compare differences in outcomes across all groups.

‡ Odd ratios and p values that are significant are bolded.

**Table S9D: Outcome odds ratios by patient “history indicative of potentially abnormal hemostasis” in all vascular surgery patients (**n=83,101)

| Outcome Variables | No. of patients | No history*  (n=64,471) | History*  (n=18,630) | Odds Ratio  (95% CI) | P-Value | Sensitivity | Specificity |
| --- | --- | --- | --- | --- | --- | --- | --- |
| Perioperative RBC transfusion | 5,519 | 3,399 (5.3%) | 2,120 (11.4%) | **2.3 (2.2-2.4)** | **<0.001** | 0.38 | 0.79 |
| Return to the OR | 4,855 | 3,291 (5.1%) | 1,564 (8.4%) | **1.7 (1.6-1.8)** | **<0.001** | 0.32 | 0.78 |
| Mortality | 971 | 496 (0.8%) | 475 (2.6%) | **3.4 (3.0-3.8)** | **<0.001** | 0.49 | 0.78 |
| Unplanned readmission | 2,484 | 1,655 (7.9%) | 829 (13.3%) | **1.8 (1.6-2.0)** | **<0.001** | 0.33 | 0.78 |

Definitions: No, number; CI = confidence interval; RBC = red blood cell; OR = operating room

* History = History indicative of potentially abnormal hemostasis

† Odd ratios and p values that are significant are bolded.

**Table S9E: Abnormal screening test odds ratios by patient “history indicative of potentially abnormal hemostasis” in vascular surgery patients screened with all 3 hemostasis tests** (n=50,839)

| Test Findings | No. of patients | No history*  (n=38,191) | History*  (n=12,648) | Odds Ratio  (95% CI) | P-Value |
| --- | --- | --- | --- | --- | --- |
| Mildly abnormal INR | 4,976 | 2,679 | 2,297 | **2.9 (2.8-3.1)** | **<0.001** |
| Severely abnormal INR | 167 | 104 | 63 | **1.8 (1.3-2.5)** | **<0.001** |
| All abnormal INR | 5,143 | 2,783 | 2,350 | **2.9 (2.8-3.1)** | **<0.001** |
| Mildly abnormal aPTT | 6,667 | 4,139 | 2,528 | **2.1 (1.9-2.2)** | **<0.001** |
| Severely abnormal aPTT | 1,585 | 767 | 818 | **3.4 (3.1-3.7)** | **<0.001** |
| All abnormal aPTT | 8,252 | 4,906 | 3,346 | **2.4 (2.3-2.6)** | **<0.001** |
| Abnormal low platelet count | 5,043 | 3,473 | 1,570 | **1.4 (1.3-1.5)** | **<0.001** |
| Abnormal high platelet count | 1,339 | 886 | 453 | **1.6 (1.4-1.8)** | **<0.001** |

Definitions: No, number; aPTT = activated partial thromboplastin time; CI = confidence interval; INR = International Normalized Ratio; OR = operating room; RBC = red blood cell

* History = History indicative of potentially abnormal hemostasis

† Odd ratios and p values that are significant are bolded.

**Table S9F: Predictive value of “patient history indicating potentially abnormal coagulation”, abnormal hemostatic test results, both, and neither in vascular surgery patients screened with all 3 hemostatic tests** (n=50,839)

| Outcome Variables | No. of patients | History* | >1 abnormal test | With history* and/or >1 abnormal test | Without history* and no abnormal coagulation tests |
| --- | --- | --- | --- | --- | --- |
| No. of patients |  | 12,648 | 15,061 | 22,219 | 28,620 |
| Perioperative RBC transfusion | 4,231 | 39.1% | 48.0% | 64.4% | 35.6% |
| Return to the OR | 3,394 | 34.5% | 41.5% | 56.0% | 44.0% |
| Mortality | 719 | 50.8% | 60.2% | 76.4% | 23.6% |
| Unplanned readmission | 1,619 | 33.9% | 38.9% | 55.6% | 44.4% |

Definitions: No, number.

* History = History indicative of potentially abnormal hemostasis
