# Supplementary material for: Use and Utility of Hemostatic Screening in Adults Undergoing Elective, Non-Cardiac Surgery
Source: PLoS One. 2015 Dec 1;10(12):e0139139. doi: 10.1371/journal.pone.0139139 (PMC4666643; doi:10.1371/journal.pone.0139139)
Supplement: S1 Text — (DOCX) [file pone.0139139.s010.docx]

**S1 Text**

**Introduction**

As described in the main text, we examined a large population of patients undergoing elective, non-cardiac surgery, across a spectrum of surgical disciplines and levels of operative risk, to assess several questions. In this supplement, we assess whether laboratory testing or patient variables may indicate dysfunctional hemostasis as an harbinger of peri-operative complications and outcome after elective, non-cardiac surgery across a variety of surgical disciplines and whether this differs between surgical conditions.

**Methods**

***Data Source:*** This study evaluated the medical records of all patients who underwent neurosurgery and were included in the American College of Surgeon (ACS) National Surgical Quality Improvement Program (NSQIP) database between 2006 and 2012. Detailed description of the ACS-NSQIP database, including design, sampling strategy, and variable definitions can be found elsewhere^1-4^. All data was collected prospectively using a standardized protocol, which consists of strictly defined variables. Each participating site had a trained, surgical nurse reviewer who was responsible for accurate and reproducible data collection from computerized and paper patient medical records, physician office records, and telephone interviews with the patient. The ACS-NSQIP database has been validated for accuracy and reproducibility^1-4^.

***Subjects:*** For the entire study population, as reported in the main text, we identified 2,302,079 adult patients who underwent and elective, non-cardiac surgery between 2006 and 2012 (Figure 1, main text; see also Methods section, main text). We excluded patients who underwent an emergency operation (n=267,285), patients diagnosed with sepsis (n=2,883), and those who received preoperative transfusion (n=11,378) . The final, global sample from which

to perform specialty-specific analyses consisted of 2,020,533 patients.

For the sub-analyses, performed individually by surgical specialty, we limited our samples in each group to the top ten procedures (by primary CPT code as the identifying and definitive procedure performed) carried out to treat the top ten diagnoses (by ICD-9 criteria). This narrowed the total subspecialty population of interest to 593,519 patients (29.4% of the starting population; Figure 1, main text), who represent the surgical patients undergoing the most common procedures for the most common diagnoses and who are most frequently seen and treated in a typical practice. The range of patients for whom a resident was present in the OR (a surrogate for a teaching facility) ranged from 26.6% and 30.7% for orthopedics and otolaryngology to 58.7% and 59.4% for vascular and general surgery, again, likely consistent with a reasonably normal distribution of surgical types and practice settings for these common procedures (see Supplemental Table 1 in each individual subspecialty for further details).

***Assessments and Statistical Analyses:*** In this supplement, general demographics, predictor variables, including hemostatic screening laboratory values and history covariables, the outcomes of interest, and statistical analyses were the same as those used for the entire dataset as a whole and are described in detail in the Methods section of the main text.

**Results**

As noted above, we limited analysis in each of the nine surgical specialties to the top ten most common surgical procedures and diagnoses to enhance the utility of the investigation for everyday practice (Table 1; and Supplemental Tables, first table in each specialty). The subgroup analyses included 593,519 patients, with the following distribution: general surgery, 48.9% of total; gynecology, 5.5%; neurosurgery, 4.1%; orthopedics, 15.3%; otolaryngology, 2.5%; plastic surgery, 2.6%; thoracic surgery, 1.3%; urology, 5.8%; and vascular surgery, 14.0% (Supplemental Table 1, each specialty).

In the general surgery cohort (n=289, 982; 48.9% of the entire sub-population), 27.3% had a PT/INR, 23.6% had an aPTT, and 76.4% had a platelet count, with 22.7% having all three tests and 22.8% having none of them; 4.7% of the patients had a composite history indicative of potentially abnormal hemostasis (“composite history” or “positive history”). Of the outcomes of interest, there were 340 deaths (0.1%), unplanned return to the OR in 6,887 (2.4%), unplanned re-admission to the hospital in 3,999 (1.4%), and 1,278 (0.4%) received a transfusion. Between 92.3% and 93.8% of the patients’ results on each of the three tests were in the normal range and for those with abnormalities, most were mild. Patients with abnormal tests had a slightly higher incidence of the outcomes of interest than did those with normal labs, but the sensitivity of a positive result was consistently low (Supplemental Table 2, general surgery). In patients who had all three tests (n=65,784 or 22.7% of the population), the number of abnormal preoperative hemostatic tests was associated with each adverse outcome, whether it was one or 2-3 abnormal tests, compared with those whose test results were in the normal range (Supplemental Table 3, general surgery); similar, and slightly more pronounced results, however, were seen in patients with a “positive history,” although sensitivities were low and specificities quite high (0.95-0.96), as seen in Supplemental Table 4, general surgery. When one examines the 65,748 patients who had all three hemostatic tests preoperatively, and the absence or presence of a “positive history,” one finds that the presence of a positive history is significantly associated with each abnormal test finding (Supplemental Table 5, general surgery). Finally, we assessed the predictive value of a “positive history”, compared with hemostatic screening, based on the percentage of each of the outcomes of interest (Supplemental Table 6, general surgery). The percentage of patients identified, with each of the outcomes, was roughly the same when using a positive history alone compared with the presence of one or more abnormal screening test alone. Even the combination of positive history plus one or more abnormal test detected only between 27.2% and 52.7% of patients with any one of the outcomes of interest. The remainder of the patients who had one of the suboptimal outcomes of interest- between just under half to almost three-quarters – was not identified by either a positive history or pre-operative hemostatic testing.

In the gynecology cohort (n=33,235; 5.5%), 22.8% had a PT/INR, 21.2% had an aPTT, and 92.1% had a platelet count, with 20.5% having all three tests and 7.7% having none of them; 2.8% of the patients had a “positive history.” Of the outcomes of interest, there were 21 deaths (0.06%), unplanned return to the OR in 456 (1.4%), unplanned re-admission to the hospital in 677 (3.5%), and 1,026 (3.1%) received a transfusion (Supplemental Table 1, gynecology). Between 95.3% and 97.3% of the patients’ results on each of the three tests were in the normal range and for those with abnormalities, most were mild. Patients with abnormal tests had a higher incidence of the outcomes of interest than did those with normal labs, but the sensitivity of a positive result was consistently low, while the sensitivity was high (Supplemental Table 2, gynecology). In patients who had all three tests (n=6,814 or 20.5% of the population), the number of abnormal preoperative hemostatic tests was associated with each adverse outcome, whether it was one or 2-3 abnormal tests, compared with those whose test results were in the normal range, although the odds ratios crossed the value of 1.0 in half or more of the results (Supplemental Table3, general surgery); more pronounced results, however, were seen in patients with a “positive history,” although sensitivities were low and specificities were high (0.97), as seen in Supplemental Table 4, gynecology. When one examines the 6,814 patients who had all three hemostatic tests preoperatively, and the absence or presence of a “positive history,” one finds that the presence of a positive history is significantly associated with each abnormal test finding, although small numbers in some cases (Supplemental Table 5, gynecology). Finally, we assessed the predictive value of a “positive history”, compared with hemostatic screening, based on the percentage of each of the outcomes of interest (Supplemental Table 6, gynecology). The percentage of patients identified, with each of the outcomes, was roughly the same when using a positive history alone compared with the presence of one or more abnormal screening test alone. Even the combination of positive history plus one or more abnormal test detected between 25.5% and 46.2% of patients with any one of the outcomes of interest. The remainder of the patients who had one of the suboptimal outcomes of interest- between just over half to almost three-quarters – was not identified by either a positive history or pre-operative hemostatic testing.

In the neurosurgery cohort (n=24,453; 4.1%), 66.7% had a PT/INR, 60.5% had an aPTT, and 90.5% had a platelet count, with 59.3% having all three tests and 8.7% having none of them; 8.9% of the patients had a “positive history.” Of the outcomes of interest, there were 77 deaths (0.3%), unplanned return to the OR in 588 (2.4%), unplanned re-admission to the hospital in 726 (3.0%), and 1,071 (4.4%) received a transfusion (Supplemental Table 1, neurosurgery). Between 94.5% and 98.1% of the patients’ results on each of the three tests were in the normal range and for those with abnormalities, most were mild. Patients with abnormal tests had a higher incidence of the outcomes of interest than did those with normal labs, but the sensitivity of a positive result was consistently low (0.02-0.16), while the sensitivity was high (0.98 in all cases; Supplemental Table 2, neurosurgery). In patients who had all three tests (n=14,500 or 59.3% of the population), the number of abnormal preoperative hemostatic tests was associated with each adverse outcome, whether it was one or 2-3 abnormal tests, compared with those whose test results were in the normal range (Supplemental Table 3, neurosurgery); more pronounced results, however, were seen in patients with a “positive history,” although sensitivities were low (range, 0.13-0.57) and specificities were high (0.91), as seen in Supplemental Table 4, gynecology. When one examines the 14,500 patients who had all three hemostatic tests preoperatively, and the absence or presence of a “positive history,” one finds that the presence of a positive history is significantly associated with each abnormal test findings in 7 of 8 instances, with stronger associations, although small numbers in some cases (Supplemental Table 5, neurosurgery). Finally, we assessed the predictive value of a “positive history”, compared with hemostatic screening, based on the percentage of each of the outcomes of interest (Supplemental Table 6, neurosurgery). The percentage of patients identified, with each of the outcomes, was roughly the same when using a positive history alone compared with the presence of one or more abnormal screening test alone. Even the combination of positive history plus one or more abnormal test detected between 23.9% and 75.4% of patients with any one of the outcomes of interest. The remainder of the patients who had one of the suboptimal outcomes of interest - from one-quarter, in the case of mortality, the least common event, and roughly three quarters for the other three outcomes - was not identified by either a positive history or pre-operative hemostatic testing.

In the orthopedic surgery cohort (n=90,627; 15.3%), 63.2% had a PT/INR, 46.7% had an aPTT, and 90.7% had a platelet count, with 45.7% having all three tests and 8.0% having none of them; 5.5% of the patients had a “positive history.” Of the outcomes of interest, there were 274 deaths (0.3%), unplanned return to the OR in 1,233 (1.4%), unplanned re-admission to the hospital in 2,295 (2.5%), and 11,968 (13.2%) received a transfusion (Supplemental Table 1, orthopedic surgery). Between 94.1% and 96.3% of the patients’ results on each of the three tests were in the normal range and for those with abnormalities, most were mild. Patients with abnormal tests had a higher incidence of the outcomes of interest than did those with normal labs, but the sensitivity of a positive result was consistently low (0.0.05-0.17), while the sensitivity was high (0.96-0.97; Supplemental Table 2, orthopedic surgery). In patients who had all three tests (n=41,445 or 45.7% of the population), the number of abnormal preoperative hemostatic tests was associated with each adverse outcome, whether it was one or 2-3 abnormal tests, compared with those whose test results were in the normal range (Supplemental Table 3, orthopedic surgery); more pronounced results, however, were seen in patients with a “positive history,” although sensitivities were low (range, 0.08-0.27) and specificities were high (0.95-0.96), as seen in Supplemental Table 4, orthopedic surgery. When one examines the 41,445 patients who had all three hemostatic tests preoperatively, and the absence or presence of a “positive history,” one finds that the presence of a positive history is significantly associated with each abnormal test finding and outcome in all cases (Supplemental Table 5, orthopedic surgery). Finally, we assessed the predictive value of a “positive history”, compared with hemostatic screening, based on the percentage of each of the outcomes of interest (Supplemental Table 6, orthopedic surgery). The percentage of patients identified, with each of the outcomes, was generally similar when using a positive history alone compared with the presence of one or more abnormal screening test alone. Even the combination of positive history plus one or more abnormal test detected between 22.4% and 49.4% of patients with any one of the outcomes of interest. The remainder of the patients who had one of the outcomes of interest which surgeons wish to avoid - from just over half to more than three-quarters of the entire group- was not identified by either a positive history or pre-operative hemostatic testing.

In the otolaryngology cohort (n=14,706; 2.5%), 30.6% had a PT/INR, 28.4% had an aPTT, and 63.3% had a platelet count, with 27.2% having all three tests and 35.8% having none of them; 5.5% of the patients had a “positive history.” Of the outcomes of interest, there were 5 deaths (0.03%), unplanned return to the OR in 362 (2.5%), unplanned re-admission to the hospital in 265 (1.8%), and 9 (0.06%) received a transfusion (Supplemental Table 1, otolaryngology). Between 95.2% and 98.0% of the patients’ results on each of the three tests were in the normal range and for those with abnormalities, most were mild. Patients with abnormal tests had a higher incidence of the outcomes of interest than did those with normal labs, but the sensitivity of a positive result was consistently low (0.0-0.6), due to low numbers, while the sensitivity was high (0.98; Supplemental Table 2, otolaryngology). In patients who had all three tests (n=4,006 or 27.2% of the population), the number of abnormal preoperative hemostatic tests was associated with each adverse outcome, whether it was one or 2-3 abnormal tests, compared with those whose test results were in the normal range, although small numbers of events limit the analysis (Supplemental Table 3, otolaryngology); more pronounced results, however, were seen in patients with a “positive history,” although sensitivities were low (range, 0.05-0.22) and specificities were high (0.97), as seen in Supplemental Table 4, otolaryngology. When one examines the 4,006 patients who had all three hemostatic tests preoperatively, and the absence or presence of a “positive history,” one finds that the presence of a positive history is similarly, and slightly more strongly associated with an abnormal test finding in the majority of cases, although small numbers limit some of the analysis (Supplemental Table 5, otolaryngology). Finally, we assessed the predictive value of a “positive history”, compared with hemostatic screening, based on the percentage of each of the outcomes of interest (Supplemental Table 6, orthopedic surgery). Here, since we are limited by small numbers in the area of mortality and blood transfusion, where the only two patients in each group had neither a “positive history” nor an abnormal hemostasis test, it is difficult to draw quantitative conclusions, neither history nor lab testing, nor a combination of the two, appeared fruitful as a pre-operative indicator of the incidence of complications.

In the plastic surgery cohort (n=15,399; 2.6%), 21.5% had a PT/INR, 19.6% had an aPTT, and 64.9% had a platelet count, with 18.8% having all three tests and 34.5% having none of them; 3.6% of the patients had a “positive history.” Of the outcomes of interest, there were 4 deaths (0.03%), unplanned return to the OR in 484 (3.1%), unplanned re-admission to the hospital in 319 (2.1%), and 187 (1.2%) received a transfusion (Supplemental Table 1, plastic surgery). 95.6% and 98.0% of the patients’ results on each of the three tests were in the normal range and for those with abnormalities, nearly all were mild. Patients with abnormal tests had a higher incidence of the outcomes of interest than did those with normal labs, but the sensitivity of a positive result was consistently low (0.0-0.07), while the sensitivity was high (0.98; Supplemental Table 2, plastic surgery). In patients who had all three tests (n=2,892, or 18.8% of the population), the number of abnormal preoperative hemostatic tests was associated with each adverse outcome, whether it was one or 2-3 abnormal tests, compared with those whose test results were in the normal range, although for one test, the odds ratio cross 1 in all cases or was not calculable (Supplemental Table 3, plastic surgery); similar results were seen in patients with a “positive history,” although the analysis is limited by the low incidence of death), as seen in Supplemental Table 4, plastic surgery. When one examines the 2,892 patients who had all three hemostatic tests preoperatively, and the absence or presence of a “positive history,” one finds that the presence of a positive history is significantly associated with abnormal test findings in roughly half the cases; the odds ratio includes a range across 1.0 in 4 of the 8 comparisons (Supplemental Table 5, plastic surgery). Finally, we assessed the predictive value of a “positive history”, compared with hemostatic screening, based on the percentage of each of the outcomes of interest (Supplemental Table 6, orthopedic surgery). The percentage of patients identified, with each of the outcomes, was generally similar when using a positive history alone compared with the presence of one or more abnormal screening test alone. Here, since we are again limited by small number, it is difficult to draw quantitative conclusions, although most patients (69.8-100%, depending upon the outcome) have neither a “positive history” nor any test abnormality.

In the thoracic surgery cohort (n=7,758; 1.3%), 77.1% had a PT/INR, 67.0% had an aPTT, and 97.1% had a platelet count, with 66.2% having all three tests and 2.3% having none of them; 19.8% of the patients had a “positive history.” Of the outcomes of interest, there were 135 deaths (1.7 %), unplanned return to the OR in 390 (5.0%), unplanned re-admission to the hospital in 482 (6.2%), and 335 (4.3%) received a transfusion (Supplemental Table 1, thoracic surgery). Between 89.3% and 94.5% of the patients’ results on each of the three tests were in the normal range and for those with abnormalities, most were mild. Patients with abnormal tests had a higher incidence of the outcomes of interest than did those with normal labs, but the sensitivity of a positive result was consistently low (0.06-0.22), while the sensitivity was high (0.95; Supplemental Table 2, thoracic surgery). In patients who had all three tests (n=5,139 or 66.2% of the population), the number of abnormal preoperative hemostatic tests was associated with each adverse outcome, whether it was one or 2-3 abnormal tests, compared with those whose test results were in the normal range, although in 4 of 8 comparisons, the odds ratio included 1.0 (Supplemental Table 3, thoracic surgery); similar results were seen in patients with a “positive history,” although sensitivities were low (range, 0.18-0.41) and specificities were moderately high (0.80-0.81), as seen in Supplemental Table 4, thoracic surgery. When one examines the 5,139 patients who had all three hemostatic tests preoperatively, and the absence or presence of a “positive history,” one finds that the presence of a positive history is associated with nearly every abnormal test finding (Supplemental Table 5, thoracic surgery). Finally, we assessed the predictive value of a “positive history,” compared with hemostatic screening, based on the percentage of each of the outcomes of interest (Supplemental Table 6, thoracic surgery). The percentage of patients identified, with each of the outcomes, was generally similar when using a positive history alone compared with the presence of one or more abnormal screening test alone. Even the combination of positive history plus one or more abnormal test detected between 32.7% and 64.0% of patients with any one of the outcomes of interest. The remainder of the patients who had one of the outcomes of interest surgeons wish to avoid - from just over one-third to more than two-thirds of the entire group- was not identified by either a positive history or pre-operative hemostatic testing.

In the urology cohort (n=34,258; 5.8%), 49.0% had a PT/INR, 43.1% had an aPTT, and 88.4% had a platelet count, with 41.6% having all three tests and 10.6% having none of them; 5.9% of the patients had a “positive history.” Of the outcomes of interest, there were 105 deaths (0.3 %), unplanned return to the OR in 613 (1.8%), unplanned re-admission to the hospital in 1,083 (3.2%), and 773 (2.3%) received a transfusion (Supplemental Table 1, urology). Between 91.1% and 94.7% of the patients’ results on each of the three tests were in the normal range and for those with abnormalities, nearly all were mild. Patients with abnormal tests had a higher incidence of the outcomes of interest than did those with normal labs, but the sensitivity of a positive result was consistently low (0.09-0.25), while the sensitivity was high (0.95; Supplemental Table 2, urology). In patients who had all three tests (n=14,254 or 41.6% of the population), the number of abnormal preoperative hemostatic tests was associated with each adverse outcome, whether it was one or 2-3 abnormal tests, compared with those whose test results were in the normal range (Supplemental Table 3, urology); similar results were seen in patients with a “positive history,” although sensitivities were low (range, 0.09-0.32) and specificities were high (0.94), as seen in Supplemental Table 4, urology. When one examines the 14,254 patients who had all three hemostatic tests preoperatively, and the absence or presence of a “positive history,” one finds that the presence of a positive history is associated with every abnormal test finding (Supplemental Table 5, urology). Finally, we assessed the predictive value of a “positive history,” compared with hemostatic screening, based on the percentage of each of the outcomes of interest (Supplemental Table 6, urology). The percentage of patients identified, with each of the outcomes, was slightly higher when using one or more abnormal test compared with a positive history, although the difference is not large. The combination of a positive history plus one or more abnormal test detected between 32.1 and 81.8% of patients with any one of the outcomes of interest, but was large only in cases of mortality, since in >61% of cases of transfusion, unplanned return to the OR or an unscheduled admission, the patient had neither a positive history nor an abnormal screening test.

In the vascular surgery cohort (n=83,101; 14.0%%), 70.8% had a PT/INR, 62.8% had an aPTT, and 92.6% had a platelet count, with 61.2% having all three tests and 6.2% having none of them; 22.4% of the patients had a “positive history.” Of the outcomes of interest, there were 971 deaths (1.2 %), unplanned return to the OR in 4,855 (5.8%), unplanned re-admission to the hospital in 2,484 (6.2%), and 5,519 (6.6%) received a transfusion (Supplemental Table 1, vascular surgery). Between 83.7% and 89.1% of the patients’ results on each of the three tests were in the normal range and for those with abnormalities, these abnormalities were mild in the vast majority of patients. Patients with abnormal tests had a higher incidence of the outcomes of interest than did those with normal labs, but the sensitivity of a positive result was consistently low (0.15-0.33), while the sensitivity was high but not as high as in most other specialties (0.89-0.90; Supplemental Table 2, vascular surgery). In patients who had all three tests (n=50,839 or 61.2% of the population), the number of abnormal preoperative hemostatic tests was associated with each adverse outcome, whether it was one or 2-3 abnormal tests, compared with those whose test results were in the normal range (Supplemental Table 3, vascular surgery); similar results were seen in patients with a “positive history,” although sensitivities were moderate (range, 0..32-0.49) and specificities were moderately high (0.78-0.79), as seen in Supplemental Table 4, vascular surgery. When one examines the 50,839 patients who had all three hemostatic tests preoperatively, and assess them by the absence or presence of a “positive history,” one finds that the presence of a positive history is associated with every abnormal test finding (Supplemental Table 5, vascular surgery). Finally, we assessed the predictive value of a “positive history,” compared with hemostatic screening, based on the percentage of each of the outcomes of interest (Supplemental Table 6, vascular surgery). The percentage of patients identified, with each of the outcomes, was generally similar when using a positive history alone compared with the presence of one or more abnormal screening test alone. Even the combination of positive history plus one or more abnormal test detected only between 55.6% and 76.4% of patients with any one of the outcomes of interest. The remainder of the patients who had one of the outcomes of interest that surgeons wish to avoid - from just under one-quarter to nearly one-half of the entire group - was not identified by either a positive history or pre-operative hemostatic testing.

**Discussion**

The findings of these subgroup analyses of patients undergoing common, elective procedures in each of nine, non-cardiac surgical disciplines, are similar to those in the group as a whole. Variable utilization of one or more of the hemostatic tests used as a screening test within the 90 days prior to the index surgery persists, and in some cases is more extreme: it ranges from (least frequent to most frequent across the nine subspecialties) from 21.5-77.1, 19.6-67.0, 63.3-97.1 percent of patients having a PT/INR, aPTT, or platelet count, with a range of 18.8-66.2 percent of patients having all three tests and 2.3-35.8 percent of patients having none of the three screening tests performed. The vast majority of abnormalities in any test were mild.

As was seen in the larger, global population, considered as a whole, when one narrows the analysis, in each surgical discipline, to the ten most common procedures performed for the most common diagnoses, hemostatic test screening is both common and inconstant and does not appear to be guided strictly or reproducibly by variables present on the patient’s pre-operative history and physical examination. Hemostatic test accuracy was suboptimal, with low sensitivity to predict the clinically-relevant outcomes of interest analyzed here, including perioperative transfusion, unplanned return to the operating room for a surgical procedure within 30 days of the index operation, readmission to a hospital and death within 30 days of surgery.

For this sub-analysis, we limited our samples in each group to the top ten procedures (by primary CPT code as the identifying and definitive procedure performed) carried out to treat the top ten diagnoses (by ICD-9 criteria). This narrowed the global population of interest to 593,519 patients (29.4% of the total population; see Figure 1, main text), which represents surgical patients who are commonly treated in a typical practice. It was also designed to insure that small sub-populations of patients (for example, a small number(s) of medically- and surgically-complex patients, in whom observed outcomes such as re-operation or death may be expected to be higher; for example, pancreaticoduodenectomy for pancreatic cancer); small numbers of procedures not performed commonly in any locale; or specific locations (eg, teaching hospitals) where uncommon procedures across a large geography converge and are grouped, might skew the analysis. Thus, the focus was on the most common procedures performed by most generalists who practice within each specialty. As with the entire population of patients, a history composite indicative of potentially abnormal hemostasis – a composite that contains simple elements that can be obtained from any patient who is to undergo elective but major, non-cardiac surgery – is both medically-reasonable and cost-effective. Further work, including prospective multi-center studies of specific populations of interest, may be indicated, to establish specific standards for preoperative hemostasis (or other laboratory) screening.

**Supplemental References:**

1. Khuri SF, Henderson WG, Daley J, Jonasson O, Jones RS, Campbell DA, Fink et al. Principal Site Investigators of the Patient Safety in Surgery Study: The Patient Safety In Surgery Study: Background, Study Design, and Patient Populations. *J Am Coll Surg* 204: 1089-1102, 2007.

2. Khuri SF. The NSQIP: A Frontier in Surgery. *Surgery* 138: 837-843, 2005.

3. American College of Surgeons National Surgical Quality Improvement Program. ACS NSQIP data collection overview. (Accessed March 2, 2015, at:

https:// acsnsqip.org/main/program_data_collection.asp)

4. Shiloach M, Frencher SK Jr, Steeger JE, et al. Toward robust information: data quality and inter-rater reliability in the American College of Surgeons National Surgical Quality Improvement Program. *J Am Coll Surg .* 210: 6-16, 2010.
